# Breast Adipose Tissue’s Xenobiotics and Fatty Acid Profile—A Preliminary Study in Portuguese Women with Breast Cancer

**DOI:** 10.3390/toxics14030224

**Published:** 2026-03-06

**Authors:** Sara Sousa, Paula Paíga, Bárbara Araújo, Francisca Coelho, Inês Castela, Miguel Vasques, Clara Sampaio, Marta Duarte, Ana Correia, Diana Teixeira, Diogo Pestana, Ana Faria, Cristina Delerue-Matos, Maria João Ramalhosa, Conceição Calhau, Valentina Fernandes Domingues

**Affiliations:** 1 REQUIMTE/LAQV, ISEP, Polytechnic of Porto, Rua Dr. António Bernardino de Almeida 431, 4249-015 Porto, Portugal; pcbpa@isep.ipp.pt (P.P.); cmm@isep.ipp.pt (C.D.-M.); mjr@isep.ipp.pt (M.J.R.); 2 Nutrition & Metabolism, CHRC-Comprehensive Health Research Centre, NOVA Medical School, Faculdade de Ciências Médicas, Universidade NOVA de Lisboa, Campo dos Mártires da Pátria 130, 1169-056 Lisboa, Portugalines.castela@nms.unl.pt (I.C.); diana.teixeira@nms.unl.pt (D.T.); ana.faria@nms.unl.pt (A.F.); 3 Faculdade de Ciências da Nutrição e Alimentação do Porto, Universidade do Porto, Rua do Campo Alegre 823, 4150-180 Porto, Portugal; 4 Champalimaud Research & Clinical Centre, Champalimaud Foundation, Av. Brasília, 1400-038 Lisboa, Portugal; miguel.vasques@nms.unl.pt; 5 NOVA Medical School, Faculdade de Ciências Médicas, Universidade NOVA de Lisboa, Campo dos Mártires da Pátria 130, 1169-056 Lisboa, Portugal; claramffsampaio@gmail.com; 6 São José Hospital, Centro Hospitalar Lisboa Central, Rua José António Serrano, 1150-199 Lisboa, Portugal; martaduartek@gmail.com (M.D.); apcorreia@net.sapo.pt (A.C.); 7 Nutrition & Metabolism, CINTESIS@RISE, NOVA Medical School, Faculdade de Ciências Médicas, Universidade NOVA de Lisboa, Campo dos Mártires da Pátria 130, 1169-056 Lisboa, Portugal; diogopestana@nms.unl.pt (D.P.); ccalhau@nms.unl.pt (C.C.)

**Keywords:** organochlorine pesticides, organophosphate pesticides, polychlorinated biphenyls, brominated flame retardants, organophosphate esters, polycyclic aromatic hydrocarbons, synthetic musks, endocrine disruptors, lipid profile

## Abstract

Countless environmental pollutants and xenobiotics, are widespread and linked to hazardous effects, including breast cancer. Due to their lipophilic properties, these accumulate in fat tissue, such as breast adipose tissue. However, little is still known about their combined effects and distribution within the breast microenvironment. Alterations in fatty acid metabolism can be a biomarker for cancer progression and a potential bioindicator of pollutant exposure. In this study, the fatty acid profile and levels of organochlorine and organophosphate pesticides (OCPs and OPPs), polychlorinated biphenyls (PCBs), brominated flame retardants (BFRs), organophosphate esters (OPEs), polycyclic aromatic hydrocarbons (PAHs) and synthetic musks (SMs) were measured in 48 breast adipose tissue samples from breast cancer and healthy patients (controls). Twelve xenobiotics were detected at high frequency rates, and the distribution profile of these pollutants differed between cohorts. In total, 163 correlations were identified between specific fatty acids and breast cancer patients’ data, with distinct correlation patterns between cohorts. Fatty acids show the potential to be biomarkers of the presence of lipophilic xenobiotics in the breast microenvironment; however, more studies are needed. This preliminary study is the first to analyze OPPs, OPEs, and PAHs in breast adipose tissue and report associations between xenobiotics and specific fatty acids.

## 1. Introduction

According to the World Health Organization (WHO), endocrine disruptors are substances that can interfere with the endocrine system function, leading to health consequences for the organism and its progeny [1]. Considered as xenobiotics, chemical substances foreign to the organism, these disruptors include man-made chemical pollutants such as brominated flame retardants (BFRs), organochlorine pesticides (OCPs), polychlorinated biphenyls (PCBs), polycyclic aromatic hydrocarbons (PAHs), organophosphate pesticides (OPPs), organophosphate esters (OPEs) and synthetic musks (SMs), amongst others. The chemicals mentioned are persistent and low-degradable, with biomagnification potential through the food chain and highly lipophilic compounds, thereby preferring to accumulate in fat tissues [2]. Due to its predominantly lipid composition, adipose tissue is well suited for evaluating the long-term bodily accumulation of these chemicals [3]. Chronic exposure to these pollutants has been associated with several health outcomes, such as metabolic disorders and carcinogenesis [2,3,4,5,6,7,8,9,10].

BFRs are valuable because of their fire-resistance characteristics; hence, they are commonly incorporated into products to reduce their flammability. Within this group, polybrominated diphenyl ethers are the most studied type of BFR [11]. Their harmful health effects led to restrictive legislation worldwide; as persistent chemicals, they are a continuous threat to human health and the ecosystem. Consequently, the production and use of novel brominated compounds have increased (e.g., decabromodiphenyl ethane, pentabromotoluene, hexabromobenzene, and pentabromoethylbenzene) [2].

OPEs are flame retardants used as substitutes for BFR, which are physically added to materials and consequently, through leaching, abrasion, or volatilization, released into the environment [4]. Currently, Regulation EC No. 1272/2008 [12] classifies them as a health and environmental hazard; however, it does not impose any restrictions on their usage. Moreover, OPEs are included in the European Union priority compounds list and the Human Biomonitoring for Europe (HBM4EU) initiative [6].

OCPs were extensively used until the 1970s. Due to long-range atmospheric transport, these synthetic pesticides can be detected in regions far away from the original application site. Known as persistent organic pollutants, their levels increase through biomagnification which, paired with high bioaccumulation properties, leads to the continuous increase in their concentration through the trophic chain [13,14].

OPPs are a class of pesticides currently widely used in agriculture and created to substitute OCP [7]. OPP’s persistence in the environment is shorter than OCP’s; however, its toxicity is higher, since its mechanism of action involves the inhibition of the acetylcholinesterase enzyme essential to nervous system control. Presently, European Union regulation EC No.1107/2009 prohibits the usage of chlorfenvinphos, parathion, and parathion-methyl [15].

PCBs are manufactured mixtures (209 congeners) and their non-flammability, electrical insulation, high boiling point, and chemical stability properties make them broadly used, amongst other things, as insulators in capacitors or transformers and in paint products [16,17]. However, after classification by the International Agency for Research on Cancer as a human carcinogen group 1 [18], their manufacture was banned almost worldwide in the late 1970s. These compounds are very lipophilic and have long half-lives, which promote their accumulation in the environment and biological samples [2].

PAHs are the subproducts of incomplete combustion or the pyrolysis process; their chemical structure includes at least two fused aromatic rings. Sixteen PAHs are listed as priority pollutants by the European Commission, besides being classified as possible carcinogens by the IARC. PAHs are highly persistent and lipophilic compounds, promoting their accumulation into fat tissues [2,8,9].

SMs are bioaccumulative and persistent xenobiotics, added to personal care products such as shampoos, body lotions, perfumes, and antiperspirants and to detergents [3,10]. European Regulation EC no.1223/2009 bans musk ambrette (MA), musk-tibetene and musk-moskene usage, and musk ketone (MK), musk xylene (MX), phantolide and tonalide (AHTN) usage in cosmetic products is restricted [19].

Breast cancer is one of the most diagnosed cancers in the female population worldwide, independent of economic development or ethnicity [20]. In Portugal, this is the most diagnosed cancer and the leading cause of death by cancer in women [21]. Every year, approximately 9000 new cases of breast cancer are diagnosed in Portugal [21] and 2.3 million worldwide [22]. Occupational and environmental exposure to some pollutants is within the known breast cancer risk factors [3,23,24,25]. Furthermore, 6% of total cancer deaths are believed to result from carcinogenic exposure. The combined effect of these chemicals, how xenobiotic endocrine disruptors interfere/alter cancer outcomes, and their distribution within the breast microenvironment are not yet fully understood [24,26].

Due to its high fat content, breast tissue is a good indicator of the chronic accumulation of lipophilic endocrine disruptors [3]. Lipophilic compounds present in breast adipose tissue can be released during adipose lipolysis [27], as lipophilic xenobiotic endocrine disruptors. The mixtures of these compounds may exhibit antagonistic or synergic effects, and biomarkers of exposure, particularly those with similar action mechanisms, are desirable [28]. A biomarker is a biological parameter whose measurement can indicate exposure (the level of lipophilic pollutants or metabolites), effect (alterations in a biological system), or susceptibility (the organism’s response to lipophilic pollutants) [29]. Adipose tissue lipids (including breast tissue) are already considered biomarkers for past dietary intake of fatty acids [27,30] and changes in their metabolism can be a biomarker for cancer progression [31,32]. Fatty acids are autacoid molecules essential for bioactive lipid synthesis and prevent metabolic dysfunction. However, high levels of fatty acids have been linked to diabetes and obesity, amongst other disorders [33]. Usually, fatty acid analysis is conducted in plasma; however, adipose tissue is a more assertive matrix for a long-term vision of a fatty acid profile since fatty acids are stored in adipose tissue and released to plasma during fasting. Some endocrine disruptors were shown to impair lipid metabolism [16,34]. Alterations in the fatty acid profile could be indicative of altered metabolism [29]. In fact, fatty acids have also been studied as biomarkers of lipophilic pollutant bioaccumulation, and their profile has proven a reliable bioindicator for assessing contamination levels; however, studies have focused mostly on marine environments [29,35,36,37,38,39]. Yet, it is plausible that adipose tissue fatty acids can also be biomarkers for lipophilic pollutant bioaccumulation in humans.

**Figure 1:**
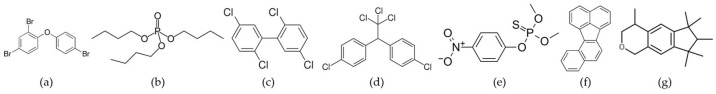
Examples of the chemical structure of the selected compound classes: (**a**) BDE28, BFR; (**b**) TnBP, OPE; (**c**) PCB52, PCB; (**d**) DDT, OCP; (**e**) parathion-methyl, OPP; (**f**) B[j]Ft, PAH and (**g**) HHCB, SM.

Aiming to address the existent gap regarding xenobiotic distribution and fatty acid composition in mammary tissue, this preliminary study quantified the fatty acid profile and concentrations of 75 endocrine disruptors (i.e., OCPs, OPPs, PCBs, BFRs, OPEs, PAHs, and SMs, Figure 1) in breast adipose tissue obtained from breast cancer and healthy patients applying a single extraction methodology. Associations between endocrine disruptors and fatty acid profile were explored to understand the potential of fatty acids as biomarkers of xenobiotic bioaccumulation. Moreover, whether endocrine disruptors were correlated with patients’ data was also assessed.

## 2. Materials and Methods

### 2.1. Chemical and Reagents

Anhydrous sodium sulphate and sodium hydroxide were purchased from Pronalab (Lisbon, Portugal); boron trifluoride–methanol (BF_3_) at 14% methanol, butylated hydroxytoluene (BHT) (≥99%) and Supelco 37 Component FAME Mix were obtained from Sigma-Aldrich (St. Louis, MO, USA); methanol was purchased from VWR Chemicals Prolabo (Fontenay-sous-Bois, Val-de-Marne, Île-de-France, France); tridecanoic acid (C13:0) was obtained from Fluka (Buchs, Switzerland) and sodium chloride (99.5%) from Panreac (Barcelona, Spain); and acetonitrile (ACN) and chromatographic-grade *n*-Hexane were purchased from Merck (Darmstadt, Germany). Elix apparatus (15.0 MΩ cm resistivity) was used to produced deionized water and a Synergy UV system (18.2 MΩ cm resistivity) from Millipore (Molsheim, France) was used to purify it into ultrapure water.

OCPs [α-, β-, γ- and δ-HCH or hexachlorocyclohexane, HCB or hexachlorobenzene, α- and β-endosulfan, aldrin, dieldrin, endrin, DDT or dichlorodiphenyltrichloroethane, DDD or dichlorodiphenyldichloroethane, DDE or 2,2-bis(p-chlorophenyl)-1,1-dichloroethene, and methoxychlor], SMs [1,3,4,6,7,8-Hexahydro-4,6,6,7,8,8-hexamethylcyclopenta[g]-2-benzopyran or HHCB (galaxolide), 4-tert-Butyl-2,6-dimethyl-3,5-dinitroacetophenone or MK (musk ketone), 4-tert-Butyl-3-methoxy-2,6-dinitrotoluene or MA (musk ambrette), 4-Acetyl-6-tert-butyl-1,1-dimethylindane or ADBI (celestolide), 2,4,6-Trinitro-1,3-dimethyl-5-tert-butylbenzene or MX (musk xylene), and 6-Acetyl-1,1,2,4,4,7-hexamethyltetralin or AHTN (tonalide)], OPEs [tripropyl phosphate or TPrP, tri-iso-butyl phosphate or TiBP, tri-n-butyl phosphate or TnBP, tris(2-chloroethyl) phosphate or TCEP, tris(2-butoxyethyl) phosphate or TBEP, tris(2-ethylhexyl) phosphate or TEHP, and tri-o-tolyl phosphate or tri-o-cresyl phosphate (TCP)], OPPs (dimethoate, chlorpyrifos-methyl, parathion-methyl, malathion, chlorpyrifos and chlorfenvinphos), and the internal standards (ISs) (1,1,1-Trichloro-2,2-bis(4-chlorophenyl)ethane or DDT d_8_, 2,2,2-trideuterio-1-(3,5,5,6,8,8-hexamethyl-6,7-dihydronaphthalen-2-yl)ethanone or AHTN d_3_, and 2,2′,4,4′5.5′-2,2′,4,4′,5,5′-hexachlorobiphenyl or PCB153 ^13^C_12_) were purchased from Sigma Aldrich (Darmstadt, Germany) and LGC Standards (Middlesex, UK).

BFRs (pentabromoethylbenzene or PBEB, pentabromotoluene or PBT, 2-ethylhexyl 2,3,4,5-tetrabromobenzoate or TBB, 2,4,4′-tribromodiphenyl ether or BDE 28, 2,2′,4,4′-tetrabromodiphenyl ether or BDE47, 2,2′,4,4′,5-pentabromodiphenyl ether or BDE99, 2,2′,4,4′,6-pentabromodiphenyl ether or BDE100, 2,2′,4,4′,5,5′-hexabromodiphenyl ether or BDE153, 2,2′,4,4′,5,6′-hexabromodiphenyl ether or BDE154, and 2,2′,3,4,4′,5′,6-heptabromodiphenyl ether or BDE183) were acquired from Isostandards Material, S.L. (Madrid, Spain).

PCBs (2,4,4′-Trichlorobiphenyl or PCB28, 2,2′,5,5′-Tetrachlorobiphenyl or PCB52, 2,2′,4,5,5′-Pentachlorobiphenyl or PCB101, 3,3′,4,4′-Tetrachlorobiphenyl or PCB77, 2,3′,4,4′,5′-Pentachlorobiphenyl or PCB118, 2,3,4,5,4′-Pentachlorobiphenyl or PCB114, 2,2′,4,4′,5,5′-Hexachlorobiphenyl or PCB153, 2,2′,3,4,4′,5′-Hexachlorobiphenyl or PCB138, 3,3′,4,4′,5′-Pentachlorobiphenyl or PCB126, 2,3,3′,4,4′,5-Hexachlorobiphenyl or PCB156, 2,3,3′,4,4′,5′-Hexachlorobiphenyl or PCB157, 2,2′,3,4,4′,5,5′-Heptachlorobiphenyl or PCB180, and 3,4,5,3′,4′,5′-Hexachlorobiphenyl or PCB169) were purchased from Techno Spec S.L. (Barcelona, Spain) and Honeywell Riedel-de Haën (Seelze, Germany).

PAHs [benzo[j]fluoranthene or B[j]Ft, dibenzo[a,l]pyrene or DB[a,l]P, and EPA 610 mixture standard with naphthalene (Naph), acenaphthylene (Acy), acenaphthene (Ace), fluorene (Flu), phenanthrene (Phe), anthracene (Ant), fluoranthene (Fln), pyrene (Pyr), benz[a]anthracene (B[a]A), chrysene (Chry), benzo[b]fluoranthene (B[b]Ft), benzo[k]fluoranthene (B[k]Ft), benzo[a]pyrene (B[a]P), dibenz[a,h]anthracene (DB[a,h]A), benzo[g,h,i]perylene (B[g,h,i]P) and indeno [1,2,3-cd]pyrene (InP)] were acquired from Supelco (Bellefonte, PA, USA).

Dispersive solid-phase extraction (SPE) 2 mL Fatty Samples AOAC and sorbet C18 Endcapped Bulk (C18EC) were acquired from Agilent technologies (Santa Clara, CA, USA); Supel QuE Z-Sep + Bulk (Z-Sep+) was purchased from Sigma-Aldrich (St. Louis, MO, USA).

Stock solutions for SM, PCB, BFR, OPP, OPE and IS solution of AHTN d_3_ and PCB153 ^13^C_12_ were prepared as described in Sousa et al. [5,40], whereas for PAH, stock solutions were prepared in ACN, and for OCP, stock solutions were prepared at 2000 µg/L in *n*-hexane. DDT d_8_ stock solutions were prepared at 5000 µg/L in *n*-hexane. Calibration curves and adipose tissue fortification were done with the stock solutions prepared, which were stored until usage at 4 °C.

### 2.2. Study Design and Sampling

Samples of breast adipose tissue were collected in 2021 at Hospital de São José (Lisbon, Portugal) Breast and Plastic Surgery units from female breast cancer patients undergoing open breast surgical procedures (hereby referred to as cases) or from female patients undergoing reduction mammoplasty (hereby referred to as controls). The Declaration of Helsinki principles were followed throughout the study and previous approval was obtained from the Centro Hospitalar Universitário Lisboa Central ethics committee (CA4664.19-3). Written informed consent was provided by all participants. Samples were kept at −80 °C until analysis. The clinical data for all participants was obtained from the Hospital’s Medical Support System.

The population under study consisted of 48 female patients, of which 42 were diagnosed with breast cancer (40 to 78 years old) and at least 62% had a hormonal breast cancer subtype. The clinical and biological characteristics of breast cancer patients are shown in Table 1. Most of the patients were from the Center of Portugal and lived in a densely or moderately populated area. Furthermore, at least 33% of breast cancer patients were overweight or obese (body mass index or BMI ≥ 25 kg/m^2^). As for tumor molecular subtype, luminal B was the most frequent (38%), followed by HER2 (12%), luminal A (10%), and triple negative (2%).

### 2.3. Fatty Acid Profile and Xenobiotic Analysis in Breast Adipose Tissue

Lipid and endocrine disruptor extraction was performed as described in the authors’ previous studies [5,8,40]. Briefly, 0.4 g of breast adipose tissue was homogenized in *n*-hexane with ultrasound-assisted extraction (UAE).

#### 2.3.1. Quantification of Endocrine Disruptors in Breast Adipose Tissue

A portion of 4.5 mL of the resulting UAE extract was dried under nitrogen and redissolved in ACN for PAH analysis, while 1.5 mL of the resulting UAE extract was purified with an AOAC dispersive solid-phase extraction (d-SPE) clean-up with additional C18EC and Z-sep+ for the analysis of the remaining endocrine disruptors.

OCP, PCB, BFR, and SM quantifications were performed, according to the authors’ previous works [40,41], by gas chromatography mass spectrometry (GC-MS). A retention time tolerance of ± 0.1 min and a ratio of selected ion’s tolerance of ±30% (relative) were enforced. NIST and Wiley libraries (similarity = 90%) and referenced standards were used to compare the mass spectra [42]. The selected ion monitoring (SIM) quantification analysis was conducted in a Trace GC Ultra gas chromatograph Polaris Q ion trap mass spectrometer (Thermo Fisher Scientific, Waltham, MA, USA), with electron impact ionization mode at 70 eV and equipped with a Zebron ZB-5MSi column (30 m × 0.25 mm and 0.25 mm film thickness, Phenomenex, Torrance, CA, USA). Xcalibur 1.3 software was used for data gathering and processing. SM and OCP analyses were conducted at a constant flow rate of 0.9 mL/min of carrier gas in splitless mode. GC oven operating conditions were as follows: initial temperature of 100 °C (1 min hold), rising at 15 °C/min to 150 °C (1 min hold), rising at 5 °C/min to 180 °C (0.5 min hold), rising at 5 °C/min to 185 °C (0.5 min hold), rising at 5 °C/min to 205 °C (0.5 min hold), rising at 5 °C/min to 225 °C (1 min hold) and finally rising at 5 °C/min to 263 °C. Transfer line, injector and ion source temperatures were kept at 265 °C, 260 °C and 270 °C, respectively. PCB and BFR analyses were conducted at a constant flow rate of 1.0 mL/min of carrier gas in splitless mode. GC oven operating conditions were as follows: initial temperature of 80 °C (1 min hold), rising at 15 °C/min to 125 °C (1 min hold), rising at 15 °C/min to 220 °C (1 min hold), rising at 10 °C/min to 230 °C (1 min hold), rising at 10 °C/min to 250 °C (1 min hold), rising at 10 °C/min to 270 °C (1 min hold) and finally rising at 10 °C/min to 290 °C (9 min hold). Transfer line, injector and ion source temperatures were kept at 270 °C, 280 °C and 292 °C, respectively. Table S1 (Supplementary Material) shows the analyte characteristics and the MS conditions [40,41].

OPP and OPE quantifications were performed according to Sousa et al. [5], using a Shimadzu GC-2010 (Kyoto, Japan) equipped with flame photometric detection (FPD) phosphorus filter, and a ZB-XLB column (30 m × 0.25 mm and 0.25 m film thickness, Zebron, Sutter Creek, CA, USA) and operated by GCSolution Shimadzu software version 2.42.00. GC oven operating conditions were as follows: initial temperature of 50 °C (1 min hold), rising at 10 °C/min to 180 °C (1 min hold), rising at 10 °C/min to 220 °C (1 min hold), rising at 10 °C/min to 275 °C (1 min hold) and finally rising at 10 °C/min to 290 °C (7 min hold). Injector and detector temperatures were kept at 250 °C and 290 °C, respectively.

Ultrapure-grade helium (purity ≥ 99.999%, Linde Sógas) was the carrier gas used in GC-MS and GC-FPD. Positives for the above-mentioned analytes were confirmed by GC tandem MS (MS/MS), using an isolation condition wideband application of 1 and the MS/MS conditions described in Supplementary Material Table S1.

PAH quantification was performed according to Sousa et al. [8], in a high-performance liquid chromatography (HPLC) system (Shimadzu LC system, Shimadzu Corporation, Kyoto, Japan) with a photodiode array (PAD SPD-M20A) and fluorescence (FLD RF-10AXL) detectors inline, using a C18 column (150 mm × 4.0 mm; 5 μm particle size; Macherey–Nagel, Duren, Germany). The LC oven temperature was set to 25 °C, and 20 µL of standard or sample was injected. Ultrapure water and ACN were the eluents used as the mobile phase. The chromatographic program in gradient mode is described in Sousa et al. [8]: 50% ACN and 50% ultrapure water (5 min hold), increasing to 100% ACN (16 min hold) and returning to the initial condition. PAHs were analyzed at excitation/emission pair wavelengths: 260/315 nm of Naph, Ace, and Flu; 260/366 nm of Phe; 260/430 nm of Ant, Fln, Pyr, B[a]A, Chry, B[b]Ft, B[j]Ft, B[k]Ft, B [a]P, DB[a,h]A, B[g,h,i]P, and DB[a,l]P; and 290/505 nm of InP and 229 nm of Acy. All PAHs were detected by FLD, except Acy. LabSolution Shimadzu software version 5.82 was used for system control and data processing.

The samples were analyzed in duplicate, and the results were corrected with average accuracy and expressed as µg/g of adipose tissue.

#### 2.3.2. Fatty Acid Analysis in Breast Adipose Tissue

The lipid content of the sample was calculated gravimetrically and expressed as g lipid/g of adipose tissue. Fatty acids were analyzed using 200 µL of the resulting UAE extract by derivatization with alkaline-catalyzed transesterification (NaOH in methanol and BF_3_) and quantified, as previously described in Sousa et al. [43], in a Shimadzu GC-2010 (Kyoto, Japan) flame ionization detector (GC-FID) in an Agilent (Santa Clara, CA, USA) J&W CP-Sil 88 capillary column (100 m × 0.25 mm I.D.; film thickness 0.20 μm), operated by GCSolution Shimadzu software version 2.42.00. BHT was used during derivatization to prevent lipid oxidation. GC analysis was conducted in split mode (1:10) and oven operating conditions were as follows: initial temperature of 100 °C (5 min hold), rising at 8 °C/min to 180 °C (9 min hold) and finally rising at 1 °C/min to 230 °C (1 min hold). Injector and detector temperatures were kept at 260 °C. The identification of fatty acid methyl esters was performed by comparing the sample chromatogram with the chromatogram of a known standard mixture (Sigma 47,885-U Supelco 37 Component FAME Mix, St. Louis, MO, USA). Examples of GC-FID chromatograms are shown in Supplementary Material Figure S1.

### 2.4. Method Validation

Method validation was conducted as described in Sousa et al. [40] for OCP analytes, with the remaining analytes being previously validated by the authors in another works [5,8,40]. Ion suppression and/or enhancement was obtained according to Paíga et al. [41]. Matrix-matched calibration curves were outlined from 5 to 350 µg/L and linearity was accepted if the coefficient of determination was ≥0.99. Method detection (MDL) and quantification (MQL) limits were calculated as the ratio of the standard deviation of the lowest calibration level to the slope of the calibration curve, multiplied by 3 and 10 for MDL and MQL, respectively.

Spiked blanks (unfortified human adipose tissue samples) were used to evaluate accuracy, in triplicate, at 20, 30, and 50 µg/L. Method repeatability and intermediate precision were determined at four levels in triplicate (5, 10, 50, and 100 µg/L). The expanded combined uncertainty (*Ur,tot*) was calculated using the “top-down” approach at 10 and 50 µg/L (95% confidence level and coverage factor k of 2), according to Nagyová et al. [44].

Matrix-matched calibration curves showed linearity (coefficient of determination > 0.99) with all analytes. MDL and MQL ranged from, respectively, 0.005 to 0.03 µg/g and 0.02 to 0.08 µg/g for SM, 0.001 to 0.03 µg/g and 0.003 to 0.08 µg/g for OCP, 0.002 to 0.04 µg/g and 0.005 to 0.1 µg/g for BFR, 0.002 to 0.02 µg/g and 0.005 to 0.06 µg/g for PCB, 0.002 to 0.009 µg/g and 0.007 to 0.03 µg/g for OPP, 0.001 to 0.006 µg/g and 0.003 to 0.02 µg/g for OPE and 0.0009 to 0.02 µg/g and 0.003 to 0.07 µg/g for PAH. Ion enhancement/suppression signals were between −26 and 23% for SM, between −30 and 12% for OCP, between −29 and 43% for BFR [40], between −33 and 21% for PCB [40], between −6 and 17% for OPP [5], between −17 and 8% for OPE [5], and between −8.1 and 7.9% for PAH [45]. Accuracies were in accordance with the European Commission guidelines [42,46], specifically: average of 82% for OCP, 91% for SM [40], 72% for BFR [40], 77% for PCB [40], 86% for OPP [5], 74% for OPE [5], and 51% for PAH [8]. Repeatability and intermediate precision ranged from, respectively, 4 to 20% and 4 to 23% for OCP, 1 to 12% and 4 to 15% for SM [40], 2 to 13% and 6 to 15% for BFR [40], 1 to 12% and 2 to 13% for PCB [40], 4 to 8% and 7 to 10% for OPP [5], 4 to 7% and 7 to 10% for OPE [5], and 0.5 to 2% and 2 to 8% for PAH [45]. Finally, *Ur,tot* was concordant with the European Commission guidelines (<50%) [42] and between 9 and 39% for OCP; between 5 and 23%, 3 and 44%, and 6 and 24%, respectively, for SM, BFR and PCB [40]; between 10 and 14% and 10 and 20% for OPP and OPE [5], respectively; and between 4 and 14% for PAH [45]. The validation parameters are detailed in Tables S2–S4 (Supplementary Material).

### 2.5. Statistical Analysis

The Statistical Package for Social Sciences (SPSS, 21.0 version, IBMCorp, New York, NY, USA) software was used to execute the statistical analysis. Data is presented as median and respective interquartile range (IQR), due to the lack of normal distribution (verified by Kolmogorov–Smirnov or by Shapiro–Wilk test if n was <50). In endocrine disruptor statistical analysis, for concentrations lower than the MDL or MQL, a value was assigned equal to the ratio of MDL or MQL to the square root of 2, respectively [47]. Median comparisons between cases and controls and between hormonal and non-hormonal breast cancer cases for endocrine disruptors, total lipids, and fatty acids were performed with the application of Mann–Whitney and Kruskal–Wallis tests. Associations between endocrine disruptors and fatty acid profile and between endocrine disruptors and case data were assessed with Spearman’s correlation test. All the tests were two-tailed and considered significant if *p* < 0.05.

## 3. Results

### 3.1. Distribution of Lipophilic Xenobiotics in Breast Adipose Tissue

**Figure 2:**
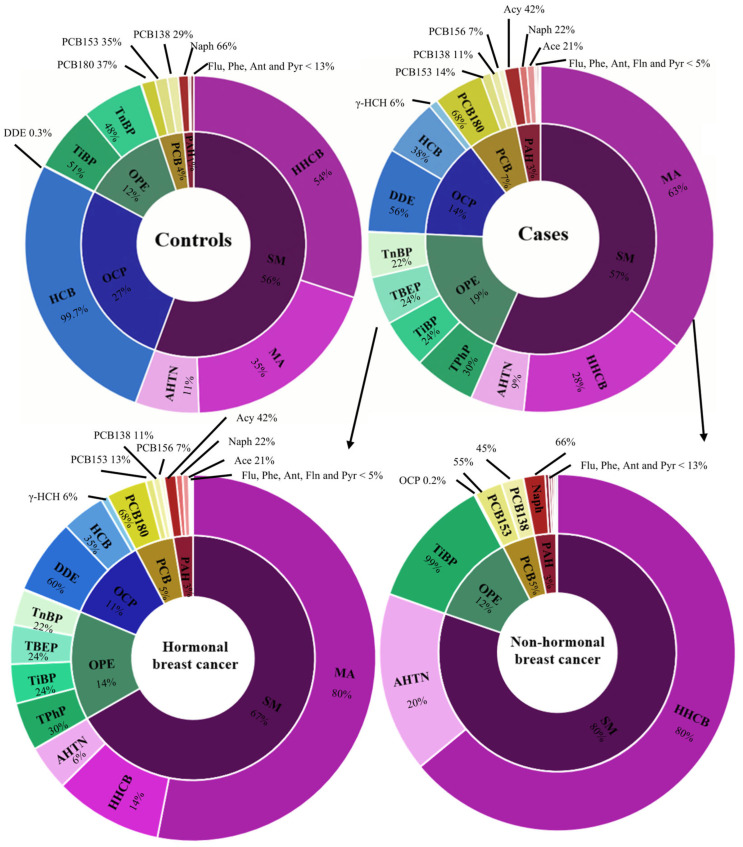
Endocrine disruptor median concentration distribution profiles in breast adipose tissue for breast cancer patients (all cases, hormonal and non-hormonal) and controls.

Three SMs, four PCBs, three OCPs, five OPEs and eight PAHs were detected in at least one breast adipose tissue sample (Table S5, Supplementary Material). BFRs and OPPs were not detected. The distribution of detected xenobiotics was different in cases and controls and different in non-hormonal breast cancer (Figure 2). Patients with hormonal breast cancer had a higher median of Naph PAH (*p* = 0.04, Table S6), and the control cohort had a higher median of TiBP OPE and Naph PAH (*p* = 0.005 and *p* = 0.01, Table S5).

HHCB and AHTN had the highest detection frequency (>93%), and concentrations ranged from <MDL to 1.9 µg/g of breast adipose tissue. As for MA, the detection frequency was below 5% and 17% for cases and controls, respectively. Concentrations ranged from 0.06 to 0.8 µg/g of breast adipose tissue, with MA being the compound with the highest median concentration for cases and hormonal breast cancer and HHCB for controls and non-hormonal breast cancer (Figure 2).

TiBP and TCP detection frequencies were higher than 93%, with TiBP being the OPE with the highest median concentration in controls and non-hormonal breast cancer. Meanwhile, TPhP had the highest median concentration for cases and hormonal breast cancer, followed by TiBP, TBEP and TnBP (Figure 2). Overall, TiBP concentration ranged from 0.04 to 0.12 µg/g of breast adipose tissue and TPhP concentration ranged from 0.05 to 0.08 µg/g of breast adipose tissue.

DDE had a detection frequency higher than 60% and concentrations between <MDL and 10.9 µg/g for cases and between <MDL and 0.3 µg/g for controls, whereas HCB and γ-HCH were detected in less than 10% of the samples tested. DDE had the highest median concentrations in cases and hormonal breast cancer, whereas HCB had the highest median concentration in controls (Figure 2).

The PCB with the highest median concentration was PCB180, followed by PCB153 and PCB138 (Figure 2) for cases, hormonal breast cancer and controls. PCB153 and PCB138 detection frequencies were higher than 30%, and PCB180 was detected in 17% and 33% of the samples for cases and controls, respectively, whereas PCB156 was only detected in one breast cancer patient.

Naph, Ace, Flu, Phe and Ant had detection frequencies higher than 80%, with Naph having the highest median concentration in controls and non-hormonal breast cancer. Acy had the highest median concentration for all cases and hormonal breast cancer, yet its detection frequency was 29% (detected at concentrations up to 0.05 µg/g of breast adipose tissue).

### 3.2. Fatty Acid Profile in Breast Adipose Tissue

The fatty acid profile and total lipid concentration for cases and controls and for hormonal and non-hormonal breast cancer cases are shown in Table 2 and Table S7, respectively. The average total lipid content of breast adipose tissue was 91% for cases and 97% for controls. The main fatty acids were as follows: C18:1ω9 *cis* (oleic acid), C16:0 (palmitic acid), and C18:2ω6 *cis* (linoleic acid or LA). C22:0, C14:1ω5, C16:1ω7, C22:1ω9 and C18:3ω6 were higher in breast adipose tissue of controls, while C17:0, C18:3ω3 (α-linolenic or ALA), C22:6ω3 *cis* (DHA) and ω3 were higher in breast adipose tissue of cases. Regarding hormonal and non-hormonal breast cancer (Table S7, Supplementary Material), C22:1ω9 and ω3 were higher in non-hormonal cases while C24:1ω9, PUFA and ω6 were higher in hormonal cases. The ratio of ω6/ω3 was lowest in breast adipose tissue of cases (16 vs. 19) and this ratio was highest in hormonal cases (16 vs. 13). The fatty acids C4:0, C6:0, C8:0, C11:0, C21:0, C23:0, C24:0, C15:1ω5 *cis*, C17:1ω7 *cis*, C18:1ω9 trans, C18:2ω6 trans and C22:2ω6 *cis* were not detected in any of the samples tested.

### 3.3. Associations Between Lipophilic Xenobiotics and Fatty Acid Profile

**Figure 3:**
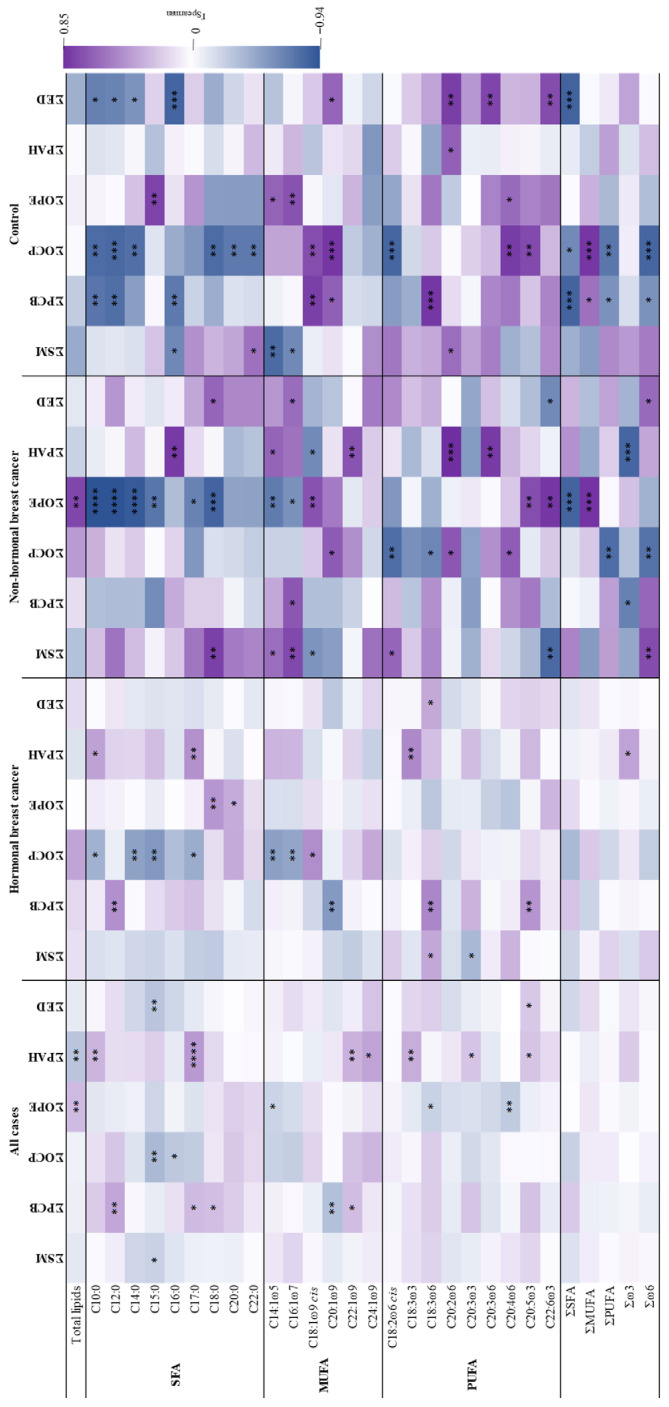
Heatmap of the associations found between endocrine disruptors and the fatty acid profile in breast adipose tissue from breast cancer patients (all cases, hormonal and non-hormonal breast cancer) and controls. The color-graded code relates to Spearman’s correlation coefficient (r_Spearman_): the lower values are in blue, and the higher values are in purple. * *p* ≤ 0.05, ** *p* ≤ 0.01, *** *p* ≤ 0.001, and **** *p* ≤ 0.0001.

Spearman correlations between fatty acids and endocrine disruptors in breast adipose tissue are described below and shown in Figure 3 and Tables S8–S11 (Supplementary Material).

Cases and controls presented different patterns of correlations (Figure 3) and within cases, hormonal and non-hormonal breast cancer displayed distinct correlations. Total lipids showed a negative correlation with the sum of PAHs in cases and positive correlations with the sum of OPEs in cases and non-hormonal breast cancer, while none was observed in controls.

Concerning SFAs, negative correlations were found with C16:0 (in controls) and C15:0 (in cases) with SM and the sum of endocrine disruptors. Furthermore, the SFA C22:0 also had a positive correlation with SM in controls. In non-hormonal cases, C18:0 had positive correlations with SM and the sum of endocrine disruptors and negative correlations were found between C10:0, C12:0, and C14:0 and the sum of endocrine disruptors. Regarding PCBs, C10:0, C12:0, and C16:0 also showed negative correlations in controls; however, in cases, C12:0, C17:0, and C18:0 achieved positive correlations. The positive correlation with C12:0 was also found in the hormonal breast cancer cohort. C10:0, C12:0, C14:0, C18:0, C20:0, and C22:0 presented negative correlations with OCP in controls while in cases, negative correlations were found with C15:0 and C16:0, and in hormonal breast cancer, negative correlations were achieved with C10:0, C14:0, C15:0 and C17:0. However, OPE achieved positive correlations with C15:0 (in controls), C18:0 and C20:0 (in hormonal breast cancer) and negative correlations with C10:0, C12:0, C14:0, C15:0, C17:0 and C18:0 (in non-hormonal breast cancer). Positive correlations were found between PAH and C10:0 and C17:0 (in cases), C18:0 and C20:0 (in hormonal breast cancer) and C16:0 (in non-hormonal breast cancer).

Regarding MUFAs, the sum of endocrine disruptors showed positive correlations with C16:1ω7 and C20:1ω9 *cis* in non-hormonal breast cancer and in controls, respectively. SM showed positive correlations with C14:1ω5 and C16:1ω7 and negative correlations with C18:1ω9 *cis* in non-hormonal breast cancer cases and negative correlations with C14:1ω5 and C16:1ω7 in controls. PCB presented positive correlations with C18:1ω9 *cis* and C20:1ω9 *cis* in controls, with C22:1ω9 in cases, and with C16:1ω7 in non-hormonal breast cancer and negative correlations with C20:1ω9 *cis* in cases and hormonal breast cancer. OCP in hormonal breast cancer had negative correlations with C14:1ω5 and C16:1ω7 and a positive correlation with C18:1ω9 *cis*. A positive correlation was also achieved between C20:1ω9 *cis* and OCP in non-hormonal breast cancer and with C18:1ω9 *cis* and C20:1ω9 *cis* in controls. Concerning OPE, positive correlations were achieved with C14:1ω5 and C16:1ω7 in controls and C18:1ω9 *cis* in non-hormonal breast cancer, whereas negative correlations were found with C14:1ω5 and C16:1ω7 in non-hormonal breast cancer. Positive correlations were found between PAH and C22:1ω9 in cases and non-hormonal breast cancer and additionally with C24:1ω9 in cases.

Concerning PUFAs, the same pattern occurred, i.e., more correlations in controls than in cases, and more correlations appeared when patients were separated into hormonal and non-hormonal breast cancer subtypes. Six correlations were obtained in cases (positive correlations: C18:3ω3 and C20:3ω3 *cis* with PAH, and C20:5ω3 *cis* with PAH and the sum of endocrine disruptors; negative correlations: C18:3ω6 and C20:4ω6 with OPE), six correlations in hormonal breast cancer cases (negative correlation: C20:3ω3 *cis* with SM; positive correlations: C18:3ω6 with SM, PCB and the sum of endocrine disruptors, C20:5ω3 *cis* with PCB, and C18:3ω3 with PAH) and eleven correlations in non-hormonal breast cancer cases (positive correlations: C18:2ω6 *cis* with SM, C20:2ω6 *cis* with OCP and PAH, C20:3ω6 *cis* with PAH, C20:4ω6 with OCP, and C20:5ω3 *cis* and C22:6ω3 *cis* with OPE; negative correlations: C18:2ω6 *cis* and C18:3ω6 with OCP, and C22:6ω3 *cis* with SM and the sum of endocrine disruptors). Meanwhile, in controls, one negative (C18:2ω6 *cis* with OCP) and nine positive correlations were found (C20:2ω6 *cis* with SM and PAH; C18:3ω6 with PCB; C20:4ω6 with OCP and OPE; C20:5ω3 *cis* with OCP; and C20:2ω6 cis, C20:3ω6 *cis* and C22:6ω3 *cis* with the sum of endocrine disruptors).

As far as the sum of SFAs, MUFAs, and PUFAs (ω3 and ω6) is concerned, correlations were found only in controls, hormonal and non-hormonal breast cancer cases, which reflects the overall pattern of correlation observed with FAs individually. In controls, seven negative ones were between the sum of SFAs and PCBs, OCPs, and the sum of endocrine disruptors, and two positive ones were between the sum of MUFAs, PCBs and OCPs, whereas, in non-hormonal breast cancer cases, negative correlations were found between OCP, PUFA, and ω6 and between PCB, PAH and ω3, and positive correlations were found between ω6, SM, and the sum of endocrine disruptors. In hormonal breast cancer, only one positive correlation was achieved between PAH and ω3.

### 3.4. Associations Between Lipophilic Xenobiotics and Breast Cancer Patients’ Data

**Figure 4:**
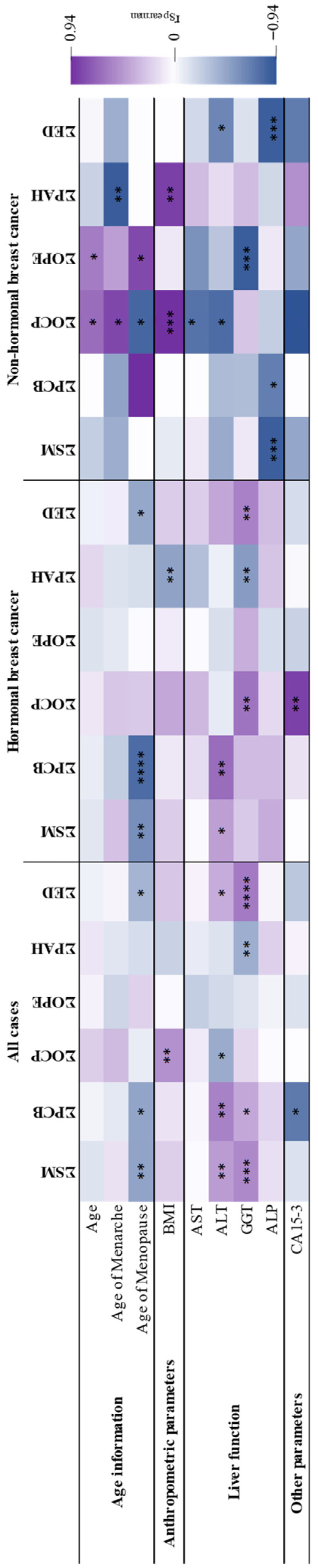
Heatmap of the associations found between endocrine disruptors in breast adipose tissue from breast cancer patients (all cases, hormonal and non-hormonal breast cancer) and the patients’ data. The color-graded code relates to Spearman’s correlation coefficient (r_Spearman_): the lower values are in blue, and the higher values are in purple. * *p* ≤ 0.05, ** *p* ≤ 0.01, *** *p* ≤ 0.001, and **** *p* ≤ 0.0001.

Spearman’s correlations between breast cancer patients’ data and endocrine disruptor levels are shown in Figure 4 and Table 3 and Table S12 (Supplementary Material).

Breast cancer patients with high blood pressure had a higher median of OCP and a lower median of PAH (Table S12).

Women under hormonal replacement therapy had a lower median of PCB and women who had prior chronic therapy had a higher median of SM (Table S12). Patients who had undergone more surgeries had a lower median concentration of PAH.

In non-hormonal breast cancer women, patient age presented positive correlations with OCP and OPE, and age of menarche achieved a positive correlation with OCP and a negative correlation with PAH (Figure 4 and Table 3). Age of menopause presented negative correlations with SM, PCB, and the sum of endocrine disruptors (Figure 4 and Table 3) in all cases and in the hormonal breast cancer subtype and with OCP in the non-hormonal breast cancer subtype. Additionally, a positive correlation was found between OPE and age of menopause in the non-hormonal breast cancer (Figure 4 and Table 3).

Overweight/obese women (BMI ≥ 25 kg/m^2^) had a higher median of OCP (Table S12), which was verified by the positive correlations between BMI, OCP and PAH (Figure 4 and Table 3). On the other hand, overweight/obese hormonal breast cancer patients showed higher medians of SM and the sum of endocrine disruptors and lower medians of PAH, supported by the negative correlation found between BMI and PAH (Figure 4 and Table 3).

Alanine aminotransferase (ALT) and gamma-glutamyl transferase (GGT) had positive correlations with SM, PCB, and the sum of endocrine disruptors (Figure 4 and Table 3) in all cases. Furthermore, these liver function parameters also showed negative correlations with OCP and PAH, respectively. In hormonal breast cancer patients, ALT also had positive correlations with SM and PCB, whereas GGT had positive correlations with OCP and the sum of endocrine disruptors and a negative correlation with PAH. On the other hand, in non-hormonal breast cancer patients, only negative correlations were found, namely: aspartate transferase (AST) with OCP, ALT with OCP and the sum of endocrine disruptors, GGT with OPE and alkaline phosphatase (ALP) with SM, and PCB and the sum of endocrine disruptors.

The tumor marker CA 15-3 had a negative correlation with PCB in cases and a positive correlation with OCP in patients with hormonal breast cancer (Figure 4 and Table 3).

## 4. Discussion

The median levels of endocrine disruptors were within the concentration range reported by other studies in breast adipose tissue, specifically up to 4.8 µg/g for OCP [3,14,20,25,28,48,49,50,51,52,53,54,55,56,57,58,59,60,61,62,63], up to 0.6 µg/g for SM [3,40,64,65,66,67,68,69,70,71] and up to 1.2 µg/g for PCB [3,17,23,24,25,28,54,56,57,61,62,63,72,73,74,75,76]. Contrary to this study, other authors report BFR in breast adipose tissue with concentrations up to 0.2 µg/g [3,24,57,63,73,74,77]. The authors did not find other studies reporting levels of OPP, OPE and PAH in human breast adipose tissue, yet some studies report concentrations of these xenobiotics in human abdominal adipose tissue (up to 0.005 µg/g for OPP [5,78,79], up to 0.1 µg/g for OPE [5,80], and up to 1.9 µg/g for PAH [8,9,81,82]).

OCP distribution was similar to other studies [3,20,25,48,50,51,62]. OCPs are known environmental estrogens and are linked to breast cancer risk [51]. However, similar median concentration of endocrine disruptors in case and control cohorts have also been reported by other authors [3,25,50]. Regarding SM, other authors report similar detection frequencies [3,40,65,66,67]. HHCB production and usage is greater than AHTN, while the usage of MA is forbidden in cosmetic products [3,67], which explains HHCB detection frequency and levels. PCB detection frequencies were also similar to those of other studies [3,17,72,74,76].

The fatty acid composition of breast adipose tissue may represent dietary intake over the past one to three years, as opposed to hours or weeks with plasma fatty acids. Furthermore, the balance of dietary intake, metabolism, and storage of fatty acids is reflected in this tissue [83]. During adipose lipolysis, the release of compounds or metabolites may occur within adipose tissue and promote tumor development/progression in breast epithelial cells [27]. Studies showed that the lipid composition of adipose tissue near a breast tumor is different from the remaining breast adipose tissue [83], thus showing the importance of assessing the breast adipose tissue fatty acid profile.

The breast adipose tissue fatty acid profile was concordant with other studies on breast adipose tissue [27,30,83,84,85,86,87] as well as the ratio ω6/ω3 [85,86]. Nonetheless, other studies reported lower levels of MUFA, DHA, and ALA and higher levels of SFA in cases than in controls (benign tumors or healthy patients) [30,85,86] or a nonsignificant difference between cases and controls in fatty acid composition [83]. A high ω6/ω3 ratio is linked to inflammation and cancer development, as ω6 inflammatory properties are opposite to ω3 anti-inflammatory ones [30].

Endocrine disruptors have long been shown to alter lipid and fatty acid metabolism and transport, for instance by promoting lipid oxidation and fatty liver syndrome [34,88,89]. For instance, PCBs have been suggested to alter lipid metabolism by interfering with nuclear transcription factors and enzyme expression, such as aryl hydrocarbon receptor (AhR), and peroxisome proliferator-activated receptor (PPAR) activation [16,90]. AhR activation has been linked to high levels of triglycerides and MUFAs, and increased fatty acid-binding protein (FABP) expression (fatty acid transport) has been linked to PCB. Furthermore, these compounds have also been linked to an increase in reactive oxygen species (ROS), which could decrease the main fatty acid oxidation pathways [16]. Endocrine disruptors can also alter the action of the fatty acid synthase enzyme present within adipose tissue, which manages the production of endogenous fatty acids. Breast adipose tissue is very active throughout a female’s lifespan, suffering alterations in hormonal stages such as puberty, pregnancy, lactation, and menopause. Additionally, the imbalance between the stroma and epithelium of hormonal signaling and growth factors can be a trigger for disease development [91]. Yet, studies associating xenobiotics with specific fatty acids are scarce.

It appears that in cases, the response to PAH triggers a lipogenesis decrease, while OPE promotes lipogenesis, which is not activated in controls. Other authors report positive correlations between MUFA and PCB, in particular with C18:1ω9 (concordant with the present study) in mink [89] and mice [92], and positive correlations with C20:5ω3 and C22:6ω3 in pregnant women [93]. Liu et al. [38] report associations between PCB, BFR and OCP and specific fatty acids in cetaceans, observing predominantly negative correlations with MUFA and PUFA (ω3 and ω6) and positive correlations with SFA. These findings are generally consistent with the correlations observed in the present study (Figure 3). Alterations in ω3 fatty acids and elevated SFA levels have been associated with chronic inflammatory diseases and increased mortality rates from coronary heart diseases [38]. Interestingly, in the present study, the significant correlations achieved between SFA and the selected xenobiotics were mainly negative and, in some instances, opposite between cohorts, highlighting the potential antagonistic or synergistic effects of mixed endocrine disruptors. In contrast to the present study, in which BFRs were not found in the analyzed samples of adipose tissue, other authors have reported correlations between BFRs and specific fatty acids in liver or blubber tissues of marine animals. Specifically, Liu et al. [38] report positive correlations with SFA and negative correlations with MUFA and PUFA, whereas Sun et al. [39] report predominantly negative correlations with SFA, MUFA and ω3 PUFA and positive correlations with ω6 PUFA.

OCP and OPE levels have been previously linked to high blood pressure [94,95,96,97] and PAH levels to lower blood pressure [98]. Hormonal replacement therapy is also a known risk factor for breast cancer development, as is endocrine disruptor exposure [25], which may explain the association found with OCP.

OCPs and OPEs are bioaccumulative; hence, they accumulate over time in the human body, explaining the association with patient age and age of menarche also reported by other authors [3,14,20,28,58,75,91,94]. Early age of menarche is a risk factor for breast cancer, particularly for hormonal breast cancer due to the hormonal and breast development changes [25,48,91,99,100]. Additionally, a later age of menarche is also linked to high mammographic density, which is, by itself, linked to breast cancer risk later on. Breast tissue with high mammographic density abounds in stromal and epithelial cells and has few adipocytes, whereas low mammographic density has high adipocytes and low stromal and epithelial cells [101]. Endocrine disruptors might have more interactions with epithelial cells in a high-mammographic-density microenvironment, subsequently increasing the susceptibility to breast cancer development. A possible explanation for the positive correlation found with non-hormonal cases was also reported by other authors on female breast cancer [28].

During menopause, metabolism changes, and there is a shift in adiposity (alteration of adipose tissue distribution) [102], subsequently leading to alteration in the breast tissue. Moreover, endocrine disruptors can also induce early menopause by damaging follicles and causing ovarian failure [103], which might be linked to the association found in this study.

The positive correlation between BMI, PAH and OCP was also reported by other studies [28,58,75]. Lipophilic pollutants can alter lipid accumulation and adipogenesis, thus acting as obesogens. In addition, it has been speculated that individuals with high BMI may have greater dietary intake of pollutants due to higher food intake [28]. Obesogens alter appetite control and energy balance as well as induce adipocyte hypertrophy and hyperplasia, leading to obesity. Furthermore, studies show that organic pollutants are capable of binding to and/or activating transcription factors such as AhR and PPARγ. AhR is important for regulating obesity- and inflammation-related genes and the metabolism of xenobiotics, while PPARγ regulates adipogenesis and the metabolism of lipids and glucose [90,91]. Moreover, Mlyczynska et al. [9] also reported negative associations with some PAHs and BMI (as in the present study for hormonal breast cancer), which may be related to the negative correlation also found with PAHs and total lipids in this study.

ALT and GGT activity seem to be altered by the studied xenobiotics. PCB and OCP show hepatotoxicity and have been previously linked to higher levels of AST, ALT, and GGT [40,104]. Furthermore, the accumulation of triglycerides in hepatocytes (associated with obesity) promotes inflammation and elevated liver function parameters (AST, ALT, GGT, and ALP) [105]. Even so, if the patients underwent neo-adjuvant therapy, liver enzymes might be altered, even though their levels appear in the normal range.

CA 15-3 marker is a glycoprotein released by breast cancer cells and usually increases as the patient’s disease progresses [106]. OCP and other xenobiotics are also linked to the risk of breast cancer progression and aggressiveness [20,24,48,107,108,109,110], which could explain the positive correlation with OCP in hormonal breast cancer patients.

**Figure 5:**
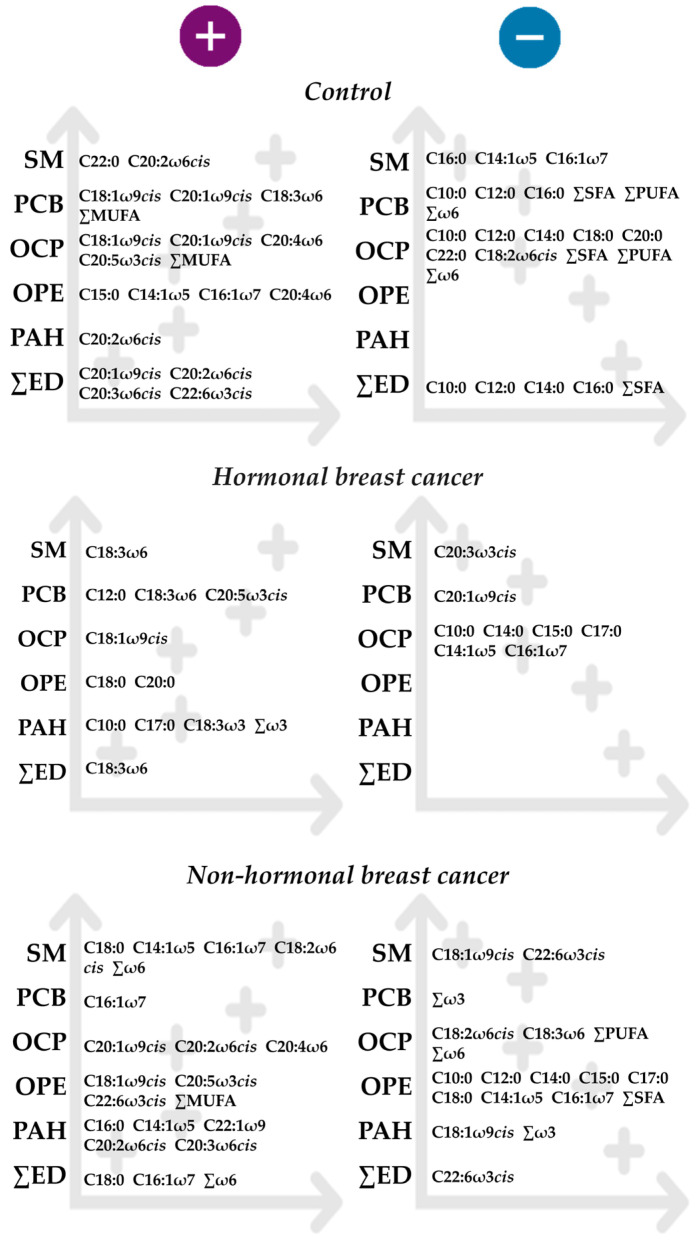
Overview of the associations between endocrine disruptors and the fatty acid profiles of breast adipose tissue in breast cancer patients and controls.

The authors acknowledge the limitations of the present study. For instance, as a preliminary study, it has a small sample size, in particular of controls and non-hormonal breast cancer cases. Breast adipose tissue sampling requires the performance of an invasive procedure, and as such, it is not an accessible matrix like blood or breast milk, which is reflected in this study and in other studies as a low sample size. Yet, in this study, 75 lipophilic endocrine disruptors were analyzed in breast adipose tissue, and the results of this preliminary study show that fatty acids and endocrine disruptor metabolism appear to be related (Figure 5). Hence, the fatty acid profile may be a biomarker of endocrine disruptor exposure. Furthermore, there is a lack of biomonitoring studies on breast adipose tissue, particularly for OPE, OPP and PAH. The present study reinforces the need to deepen research on the fatty acid breast microenvironment and its lipophilic endocrine disruptors.

## 5. Conclusions

The assessment of lipophilic xenobiotics in breast adipose tissue could bring some elucidation to breast cancer development and progression. Together with the analysis of the fatty acid profile, crucial information about the effects of endocrine-disrupting chemicals within the breast microenvironment may be achieved. The study of the breast microenvironment should not be discarded due to the proximity to tumors, among other factors. There is a lack of studies on xenobiotic and fatty acid distribution in mammary tissue. To the authors’ knowledge, this is the first study analyzing OPP, OPE, and PAH in breast adipose tissue and presenting direct associations between endocrine disruptors and specific fatty acids. A wide range of endocrine disruptors and the fatty acid profile were analyzed with a small amount of breast adipose tissue. SM, OCP, PCB, OPE, and PAH had high detection frequencies, with a total endocrine disruptor median concentration of 0.4 µg/g for cases and 0.5 µg/g for controls. Furthermore, correlations with fatty acid profile and data of breast cancer patients are distinct between case/control and hormonal/non-hormonal breast cancer, showing the possible effects of these pollutants on the human body and the importance of further studies.

## Figures and Tables

**Table 1:** Clinical and biological characteristics of breast cancer patients.

		All Cases			Hormonal Breast Cancer			Non-Hormonal Breast Cancer			Hormonal vs. Non-Hormonal Breast Cancer
		*n*	Median	IQR	*n*	Median	IQR	*n*	Median	IQR	*p*
Age information	Age (years)	26	63	10	20	63	9	6	63	18	0.632
	Menarche (years)	18	14	2	14	13	2	4	15	1	**0.001**
	Menopause (years) if applicable	14	47	26	11	45	14	4	50	12	0.910
Anthropometric parameters	BMI (kg/m^2^)	20	26	6	15	26	6	5	25	3	0.168
Liver function	AST (U/L)	17	20	7.5	12	20	6	5	17	21	0.595
	ALT (U/L)	17	17	10	12	15	8	5	24	17	**0.009**
	GGT (U/L)	18	25	27	13	24	11	5	48	22	**<0.001**
Other parameters	ALP (U/L)	18	78	27	13	78	28	5	78	35	0.435
	CA15-3 (U/mL)	13	18	21	10	17	24	3	18	23	0.464

ALP—alkaline phosphatase; ALT—alanine aminotransferase; AST—aspartate aminotransferase; BMI—body mass index; CA—cancer antigen; GGT—gamma glutamyl transpeptidase; IQR—interquartile range. Statistical analysis by Mann–Whitney and Kruskal–Wallis tests; Significant *p* values are shown in bold, *p* < 0.05.

**Table 2:** Total lipid concentration (g/g breast adipose tissue) and fatty acid profile in cases and controls (%).

	Case				Control				Case vs. Control
	n	Frequency	Median	IQR	n	Frequency	Median	IQR	*p*
Total Lipids	42	100%	0.98	0.12	6	100%	1.00	0.05	0.071
Fatty acids									
C4:0	0		nd		0		nd		
C6:0	0		nd		0		nd		
C8:0	0		nd		0		nd		
C10:0	41	98%	0.02	0.02	6	100%	0.03	0.21	0.089
C11:0	0		nd		0		nd		
C12:0	42	100%	0.3	0.2	6	100%	0.4	0.4	0.240
C14:0	42	100%	2.2	0.8	6	100%	2.1	0.5	0.723
C15:0	42	100%	0.19	0.05	6	100%	0.18	0.08	0.184
C16:0	42	100%	23	3	6	100%	22	4	0.303
C17:0	42	100%	0.21	0.05	6	100%	0.18	0.06	**0.01**
C18:0	42	100%	4	1	6	100%	4	2	0.180
C20:0	42	100%	0.10	0.06	6	100%	0.12	0.09	0.232
C21:0	0		nd		0		nd		
C22:0	42	100%	0.02	0.02	6	100%	0.03	0.03	**0.042**
C23:0	0		nd		0		nd		
C24:0	0		nd		0		nd		
ΣSFA	42	100%	30	4	6	100%	28	6	0.268
C14:1 ω5	42	100%	0.13	0.09	6	100%	0.2	0.1	**0.022**
C15:1 ω5 *cis*	0		nd		0		nd		
C16:1 ω7	42	100%	3	1	6	100%	4	3	**0.026**
C17:1 ω7 *cis*	0		nd		0		nd		
C18:1 ω9 *trans*	0		nd		0		nd		
C18:1 ω9 *cis*	42	100%	46	4	6	100%	47	6	0.565
C20:1 ω9 *cis*	42	100%	0.6	0.1	6	100%	0.6	0.2	0.756
C22:1 ω9	42	100%	0.020	0.009	6	100%	0.030	0.003	**<0.001**
C24:1 ω9	20	46%	0.010	0.007	6	100%	0.011	0.006	0.228
ΣMUFA	42	100%	50	4	6	100%	52	6	0.069
C18:2 ω6 *trans*	0		nd		0		nd		
C18:2 ω6 *cis*	42	100%	17	3	6	100%	14	8	0.166
C18:3 ω3	42	100%	0.5	0.2	6	100%	0.4	0.1	**0.006**
C18:3 ω6	42	100%	0.04	0.02	6	100%	0.06	0.02	**0.028**
C20:2 ω6 *cis*	42	100%	0.26	0.09	6	100%	0.25	0.04	0.166
C20:3 ω3 *cis*	42	100%	0.016	0.008	6	100%	0.01	0.01	0.245
C20:3 ω6 *cis*	42	100%	0.4	0.3	6	100%	0.25	0.09	0.177
C20:4 ω6	42	100%	0.5	0.3	6	100%	0.7	0.4	0.121
C20:5 ω3 *cis*	42	100%	0.12	0.07	6	100%	0.09	0.08	0.223
C22:2 ω6 *cis*	0		nd		0		nd		
C22:6 ω3 *cis*	42	100%	0.4	0.3	6	100%	0.22	0.07	**0.01**
ΣPUFA	42	100%	19	3	6	100%	16	8	0.101
Σω3	42	100%	1.1	0.4	6	100%	0.8	0.7	**0.038**
Σω6	42	100%	18	4	6	100%	15	8	0.141

IQR—interquartile range; MUFA—monounsaturated fatty acid; PUFA—polyunsaturated fatty acid; SFA—saturated fatty acid; nd—not detected; ω—omega; ω3—omega 3 fatty acids; ω6—omega 6 fatty acids. Statistical analysis performed with Mann–Whitney and Kruskal–Wallis tests; *p* < 0.05. Significant *p* values are shown in bold.

**Table 3:** Spearman’s correlation coefficient (r_S_) and *p* values between endocrine disruptors in breast adipose tissue from breast cancer patients (all cases, hormonal and non-hormonal breast cancer) and the patients’ data. The values in bold indicate significant associations (*p* < 0.05).

		ΣSM		ΣPCB		ΣOCP		ΣOPE		ΣPAH		ΣED	
		r_S_	*p*	r_S_	*p*	r_S_	*p*	r_S_	*p*	r_S_	*p*	r_S_	*p*
		**All Cases**											
Age information	Age	−0.14	0.322	−0.05	0.743	0.22	0.199	0.05	0.703	0.11	0.442	−0.07	0.636
	Age of Menarche	0.13	0.462	−0.11	0.596	0.31	0.162	−0.22	0.207	−0.13	0.462	0.04	0.801
	Age of Menopause	**−0.49**	**0.008**	**−0.49**	**0.021**	−0.09	0.701	0.20	0.310	−0.18	0.371	**−0.40**	**0.033**
Anthropometric parameters	BMI	0.21	0.185	0.12	0.497	**0.50**	**0.007**	0.02	0.915	−0.23	0.147	0.25	0.117
Liver function	AST	−0.01	0.966	0.03	0.872	0.09	0.683	−0.26	0.142	−0.09	0.605	0.05	0.774
	ALT	**0.45**	**0.008**	**0.57**	**0.003**	**−0.45**	**0.027**	−0.19	0.276	−0.15	0.414	**0.37**	**0.029**
	GGT	**0.54**	**0.001**	**0.39**	**0.031**	0.17	0.397	−0.14	0.431	**−0.44**	**0.008**	**0.60**	**<0.0001**
	ALP	0.14	0.433	0.19	0.327	−0.01	0.977	−0.05	0.783	0.21	0.229	0.05	0.788
Other parameters	CA15-3	−0.15	0.462	**−0.73**	**0.041**	0.00	1.000	−0.14	0.483	0.05	0.800	−0.30	0.140
		**Hormonal breast cancer**											
Age information	Age	−0.12	0.458	−0.09	0.639	0.11	0.604	−0.14	0.376	0.17	0.280	−0.06	0.708
	Age of Menarche	0.27	0.157	−0.26	0.262	0.25	0.349	−0.11	0.579	−0.14	0.464	0.08	0.694
	Age of Menopause	**−0.61**	**0.003**	**−0.81**	**<0.0001**	0.24	0.377	0.01	0.968	−0.17	0.444	**−0.47**	**0.027**
Anthropometric parameters	BMI	0.23	0.230	0.10	0.630	0.39	0.085	0.08	0.656	**−0.50**	**0.005**	0.22	0.234
Liver function	AST	−0.01	0.955	0.15	0.577	0.32	0.233	0.01	0.964	−0.34	0.107	0.22	0.311
	ALT	**0.43**	**0.036**	**0.67**	**0.004**	−0.10	0.719	−0.16	0.447	−0.07	0.746	0.40	0.052
	GGT	0.24	0.246	0.30	0.204	**0.64**	**0.005**	0.36	0.068	**−0.54**	**0.005**	**0.57**	**0.002**
	ALP	0.37	0.060	0.30	0.203	0.16	0.513	−0.18	0.390	0.26	0.198	0.29	0.144
Other parameters	CA15-3	0.00	1.000	0.12	0.703	**0.86**	**0.001**	−0.23	0.324	−0.02	0.939	−0.18	0.452
		**Non-hormonal breast cancer**											
Age information	Age	−0.25	0.424	0.00	1.000	**0.67**	**0.036**	**0.60**	**0.041**	−0.23	0.468	−0.03	0.931
	Age of Menarche	−0.44	0.276	−0.49	0.324	**0.84**	**0.036**	0.44	0.280	**−0.89**	**0.003**	−0.44	0.280
	Age of Menopause	0.00	1.000	0.94	0.057	**−0.84**	**0.036**	**0.83**	**0.042**	0.00	1.000	0.00	1.000
Anthropometric parameters	BMI	−0.10	0.786	0.00	1.000	**0.94**	**0.0006**	0.10	0.786	**0.87**	**0.001**	0.00	1.000
Liver function	AST	0.10	0.787	0.00	1.000	**−0.77**	**0.026**	−0.59	0.071	0.30	0.400	−0.20	0.586
	ALT	−0.45	0.187	−0.36	0.306	**−0.81**	**0.015**	−0.30	0.393	0.15	0.671	**−0.66**	**0.039**
	GGT	0.10	0.787	−0.35	0.319	0.26	0.541	**−0.89**	**0.0006**	0.30	0.400	−0.15	0.684
	ALP	**−0.89**	**0.0006**	**−0.70**	**0.023**	−0.26	0.541	0.10	0.786	−0.20	0.580	**−0.89**	**0.0006**
Other parameters	CA15-3	−0.48	0.338	0.00	1.000	−0.94	0.057	−0.49	0.329	0.50	0.312	−0.72	0.109

ALP—alkaline phosphatase; ALT—alanine aminotransferase; AST—aspartate aminotransferase; BMI—body index mass; CA—cancer antigen; GGT—gamma glutamyl transpeptidase; rs—Spearman’s correlation coefficient. Significant *p* values are shown in bold; *p* < 0.05.

## Data Availability

The original contributions presented in this study are included in the article/Supplementary Material. Further inquiries can be directed to the corresponding author.

## Supplementary Materials

The following supporting information can be downloaded at: https://www.mdpi.com/article/10.3390/toxics14030224/s1, Table S1: MS, MS/MS conditions and characteristics for SM, BFR, PCB and OCP; Figure S1: Overlay of GC-FID chromatograms of FAs: sample of breast adipose tissue spiked with internal standard (pink) and FA standard mixture (black); Table S2: Method validation parameters for OCP in breast adipose tissue spiked at different levels; Table S3: Method validation parameters for SM in breast adipose tissue spiked at different levels; Table S4: Method validation parameters for PCB, BFR and PAH in breast adipose tissue spiked at different levels; Table S5: SM, PCB, BFR, OCP and PAH concentrations in cases and controls (µg/g breast adipose tissue); Table S6: SM, PCB, BFR, OCP and PAH concentrations in hormonal and non-hormonal breast cancer (µg/g breast adipose tissue); Table S7: Total lipid concentration (g/g breast adipose tissue) and fatty acid profile in hormonal and non-hormonal breast cancer (%); Table S8: Spearman’s correlation coefficient (rS) of the associations found between endocrine disruptors and the fatty acid profile in breast adipose tissue from breast cancer patients. The values in bold indicate significant associations (*p* < 0.05); Table S9: Spearman’s correlation coefficient (rS) of the associations found between endocrine disruptors and the fatty acid profile in breast adipose tissue from controls. The values in bold indicate significant associations (*p* < 0.05); Table S10: Spearman’s correlation coefficient (rS) of the associations found between endocrine disruptors and the fatty acid profile in breast adipose tissue from hormonal breast cancer patients. The values in bold indicate significant associations (*p* < 0.05); Table S11: Spearman’s correlation coefficient (rS) of the associations found between endocrine disruptors and the fatty acid profile in breast adipose tissue from non-hormonal breast cancer patients. The values in bold indicate significant associations (*p* < 0.05); Table S12: Breast cancer patients’ data and relation with the sum of SM, PCB, OCP and PAH concentrations (µg/g breast adipose tissue).

## Abbreviations

The following abbreviations are used in this manuscript:

Ace	acenaphthene
ACN	acetonitrile
Acy	acenaphthylene
ADBI	celestolide
AhR	aryl hydrocarbon receptor
AHTN d_3_	2,2,2-trideuterio-1-(3,5,5,6,8,8-hexamethyl-6,7-dihydronaphthalen-2-yl)ethanone
AHTN	tonalide
ALA	α-linolenic
ALP	alkaline phosphatase
ALT	alanine aminotransferase
Ant	anthracene
AST	aspartate transferase
B[a]A	benz[a]anthracene
B[a]P	benzo[a]pyrene
B[b]Ft	benzo[b]fluoranthene
B[g,h,i]P	benzo[g,h,i]perylene
B[k]Ft	benzo[k]fluoranthene
BDE	bromodiphenyl ether
BF_3_	boron trifluoride–methanol
BFR	brominated flame retardant
BHT	butylated hydroxytoluene
BMI	body mass index
C18EC	C18 endcapped bulk
Chry	chrysene
DB[a,h]A	dibenz[a,h]anthracene
DB[a,l]P	dibenzo[a,l]pyrene
DDD	dichlorodiphenyldichloroethane
DDE	2,2-bis(p-chlorophenyl)-1,1-dichloroethene
DDT d_8_	1,1,1-trichloro-2,2-bis(4-chlorophenyl)ethane
DDT	dichlorodiphenyltrichloroethane
DHA	docosahexaenoic acid
ET	excitation time
EV	excitation voltage
FABP	fatty acid-binding protein
FID	flame ionization detection
FLD	fluorescence
Fln	fluoranthene
Flu	fluorene
FPD	flame photometric detection
FSH	Follicle-stimulating hormone
GC	gas chromatography
GGT	gamma-glutamyl transferase
HCB	hexachlorobenzene
HCH	hexachlorocyclohexane
HDL	high-density lipoprotein
HHCB	galaxolide
HPLC	high-performance liquid chromatography
InP	indeno [1,2,3-cd]pyrene
IQR	interquartile range
IS	internal standard
IT	isolation time
LA	linoleic acid
LDL	low-density lipoprotein
MA	musk ambrette
MDL	method detection limit
MK	musk ketone
MQL	method quantification limit
MS	mass spectrometry
MS/MS	tandem MS
MUFA	monounsaturated fatty acid
MX	musk xylene
na	not applicable
Naph	naphthalene
nd	not detected
OCP	organochlorine pesticide
OPE	organophosphorus ester
OPP	organophosphorus pesticide
PAD	photodiode array
PAH	polycyclic aromatic hydrocarbon
PBEB	pentabromoethylbenzene
PBT	pentabromotoluene
PCB	polychlorinated biphenyl
Phe	phenanthrene
PPAR	peroxisome proliferator-activated receptor
PUFA	polyunsaturated fatty acid
Pyr	pyrene
ROS	reactive oxygen species
RT	retention time
SFA	saturated fatty acid
SIM	selected ion monitoring
SM	synthetic musk
SPE	solid-phase extraction
T3	triiodothyronine
T4	thyroxine
TBB	2-ethylhexyl 2,3,4,5-tetrabromobenzoate
TBEP	tris(2-butoxyethyl) phosphate
TCEP	tris(2-chloroethyl) phosphate
TCP	tri-o-tolyl phosphate or tri-o-cresyl phosphate
TEHP	tris(2-ethylhexyl) phosphate
TiBP	tri-iso-butyl phosphate
TnBP	tri-n-butyl phosphate
TPrP	tripropyl phosphate
TSH	thyroid-stimulating hormone
UAE	ultrasound-assisted extraction
*Ur,tot*	expanded combined uncertainty
WHO	World Health Organization
Z-Sep+	Supel QuE Z-Sep + Bulk
ω3	omega 3
ω6	omega 6
