# Supplementary material for: Breast Adipose Tissue’s Xenobiotics and Fatty Acid Profile—A Preliminary Study in Portuguese Women with Breast Cancer
Source: Toxics. 2026 Mar 6;14(3):224. doi: 10.3390/toxics14030224 (PMC13030003; doi:10.3390/toxics14030224)
Supplement: Supplementary file 1 [file toxics-14-00224-s001.zip › toxics-4114943-supplementary.pdf]

Breast Adipose Tissue's Xenobiotics and Fatty Acid Profile – A Preliminary Study in Portuguese Women with Breast Cancer - Supplementary Material

Table S1. MS, MS/MS conditions and characteristics for SM, BFR, PCB, OCP, OPP, and OPE [40,41].

	Analyte	Molecular weight (g/mol)	RT (min)	MS		MS/MS				
				Quantifier ion (m/z)	Qualifier ions (m/z)	MRM transitions (m/z)	Fragmentation			
							factor "q"	IT (ms)	ET (ms)	EV (v)
SM	ABDI	244	14.26	229	43, 173, 244	229 → 173	0.45	12	15	1
	MA	268	16.78	253	91	253 → 91	0.225			
	HHCB	258	17.12	243	213, 258	243 → 213	0.45			
	AHTN	258	17.32	243	187, 258	243 → 187	0.45			
	MK	294	19.97	279	294	279 → 191	0.225			
	MX	297	17.36	282	297	282 → 265	0.225			
	AHTN-d3 (IS)	261	17.36	246	160, 190	na	na	na	na	na
BFR	BDE 28	406	17.97	406	246, 247, 248	406 → 248	0.225	12	15	1
	PBT	486	18.19	486	247, 407	486 → 326	0.45			
	PBEB	500	18.69	500	340, 421, 485	500 → 406	0.225			
	BDE 47	486	20.91	486	326, 488	486 → 326	0.30			
	BDE 100	564	23.12	406	404, 564	406 → 297	0.30			
	BDE 99	564	23.80	406	404, 564	406 → 297	0.30			
	TBB	750	23.80	419	312, 393, 421	419 → 391	0.30			
	BDE 154	644	26.16	644	484, 643	644 → 484	0.45			
	BDE 153	644	27.56	644	484, 643	644 → 484	0.45			
	BDE 183	721	33.66	561	561, 564, 720, 723	561 → 452	0.30			

ABDI – celestolide; AHTN – tonalide; BDE – bromodiphenyl ether; BFR – brominated flame retardant; ET - excitation time; EV – excitation voltage; HHCB – galaxolide; IS – internal standard; IT – isolation time; MA – musk ambrette; MK – musk ketone; MS – mass spectrometry; MS/MS – tandem MS; MX – musk xylene; na - not applicable; PBEB – pentabromoethylbenzene; PBT – pentabromotoluene; RT – retention time; SM – synthetic musk; TBB - 2-ethylhexyl 2,3,4,5-tetrabromobenzoate.

Table S1. MS, MS/MS conditions and characteristics for SM, BFR, PCB, OCP, OPP ,and OPE [40,41] (continuation).

	Analyte	Molecular weight (g/mol)	RT (min)	MS		MS/MS				
				Quantifier ion (m/z)	Qualifier ions (m/z)	MRM transitions (m/z)	Fragmentation			
							factor "q"	IT (ms)	ET (ms)	EV (v)
	PCB 28	257	13.66	256	150, 186	256 → 186				
	PCB 52	292	14.39	292	150, 220, 255	292 → 255				
	PCB 101	326	16.34	326	184, 254	326 → 256				
	PCB 77	292	17.24	292	185, 220	292 → 222				
	PCB 118	326	17.85	326	219, 254, 256	326 → 256				
	PCB 114	326	18.13	326	219	326 → 256				
PCB	PCB 153	361	18.33	360	290	360 → 290	0.45	12	15	1
	PCB 138	361	19.02	360	218, 290	360 → 290				
	PCB 126	326	19.46	326	219, 256	326 → 256				
	PCB 156	361	20.39	360	290	360 → 290				
	PCB 157	361	20.52	360	290	360 → 290				
	PCB 180	395	20.78	394	324	394 → 324				
	PCB 169	361	21.34	360	288	360 → 290				
	PCB 153 $^{13}C_{12}$ (IS)	373	18.36	372	302	na	na	na	na	na
	α-HCH	291	13.96	181	109, 219	181 → 145	0.45	12	60	1
	β-HCH	291	14.93	181	109, 219	181 → 145	0.45	12	60	1
	γ-HCH	291	15.29	183	183, 219	183 → 179	0.45	12	60	1
	δ-HCH	291	16.31	181	109, 219	181 → 145	0.45	12	60	1
	HCB	285	14.26	284	142, 286	284 → 142	0.45	2	15	0.2
	Aldrin	365	19.76	263	66, 293	263 → 191	0.45	12	60	2
	Dieldrin	381	24.13	263	79, 277	263 → 243	0.45	12	60	2
OCP	Endrin	381	25.05	263	81, 281	263 → 193	0.45	12	60	2
	α-endosulfan	407	22.98	241	195, 207	241 → 195	0.3	2	5	0.2
	β-endosulfan	407	25.46	195	207, 241	195 →170	0.3	2	15	0.2
	DDE	318	24.13	246	176, 318	246 →176	0.45	2	5	0.2
	DDT	355	25.99	235	165	235 → 165	0.45	36	60	2
	DDD	320	25.88	235	165	235 → 165	0.45	36	60	2
	Methoxychlor	346	29.82	227	237	227 → 169	0.3	12	60	2
	DDT d_8 (IS)	363	27.29	243	169, 170, 171, 245	na	na	na	na	na
	Dimethoate	229	11.13	na	na	125 → 63	0.45		15	1
	Chlorpyrifos-methyl	322	14.16	na	na	286 → 241	0.45		15	1
OPP	Parathion-methyl	263	14.40	na	na	263 → 246	0.45	12	15	1
	Malathion	330	15.84	na	na	173 → 99	0.45		5	0.2
	Chlorpyrifos	350	16.14	na	na	314 → 194	0.30		15	0.2
	Chlorfenvinphos	360	17.90	na	na	267 → 159	0.45		15	1
	TPrP	224	5.82	na	na	99 → 81				
	TiBP	266	7.39	na	na	99 → 81				
	TnBP	266	9.48	na	na	99 → 81				
OPE	TCEP	286	11.63	na	na	249 → 187	0.45	12	15	1
	TBEP	399	21.37	na	na	277 → 125				
	TPhP	326	21.42	na	na	99 → 81				
	TEHP	435	21.75	na	na	99 → 81				
	TCP	368	23.50	na	na	368 → 261				

DDD - dichlorodiphenyldichloroethane; DDE - 2,2-bis(p-chlorophenyl)-1,1-dichloroethene; DDT - dichlorodiphenyltrichloroethane; ET -excitation time; EV – excitation voltage; HCB - hexachlorobenzene; HCH - hexachlorocyclohexane; IS – internal standard; IT – isolation time; MS – mass spectrometry; MS/MS – tandem MS; na - not applicable; OCP – organochlorine pesticide; OPE - Organophosphorus ester; OPP - organophosphorus pesticide; PCB - polychlorinated biphenyls; RT – retention time; SM – synthetic musk; TBEP - tris(2-butoxyethyl) phosphate; TCEP - tris(2-chloroethyl) phosphate; TCP - tri-o-tolyl phosphate or tri-o-cresyl phosphate; TEHP - tris(2-ethylhexyl) phosphate; TiBP - tri-iso-butyl phosphate; TnBP - tri-n-butyl phosphate; TPrP - tripropyl phosphate.

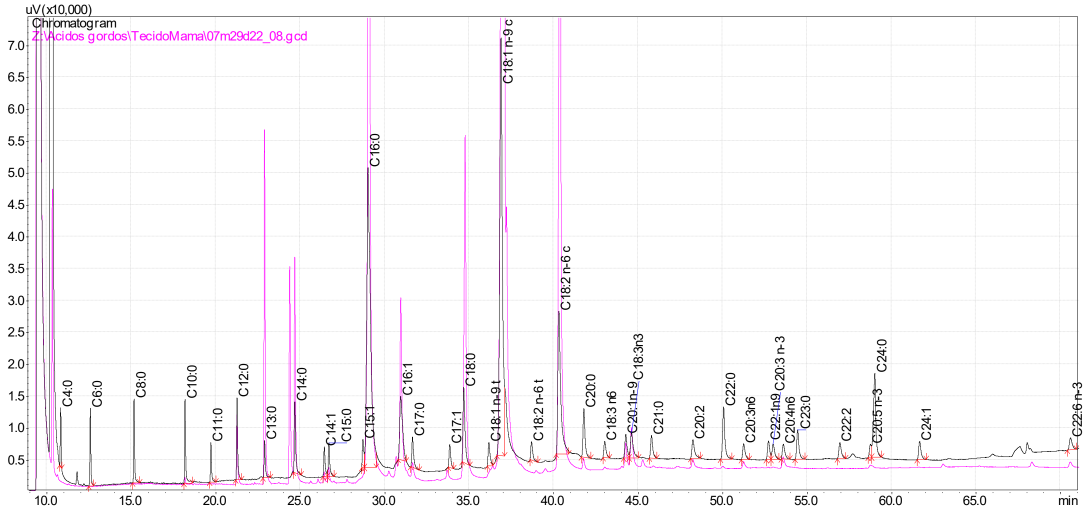

Figure S1. Overlay of GC-FID chromatograms of FAs: sample of breast adipose tissue spiked with internal standard (pink) and FA standard mixture (black).

Table S2. Method validation parameters for OCP in breast adipose tissue spiked at different levels.

	Analyte	Ion enhancement/ suppression (%)	Coefficient of determination	Accuracy (%)			Repeatability (%)				Intermediate precision (%)				MDL (μg/g)	MQL (μg/g)	*Ur,tot* (%)	
				20 μg/L	30 μg/L	50 μg/L	5 μg/L	10 μg/L	50 μg/L	100 μg/L	5 μg/L	10 μg/L	50 μg/L	100 μg/L			10 μg/L	50 μg/L
OCP	α-HCH	-26	0.990	74	98	88	11	5	12	13	16	8	12	17	0.01	0.03	38	9
	β-HCH	1	0.991	91	94	96	13	11	9	11	13	15	16	12	0.03	0.08	37	13
	γ-HCH	-24	0.985	68	76	94	11	9	13	8	20	11	14	9	0.01	0.04	34	20
	δ-HCH	-12	0.988	76	77	98	11	8	13	3	21	9	14	7	0.01	0.04	34	11
	HCB	-25	0.987	62	70	77	20	12	11	10	23	16	12	17	0.01	0.03	27	10
	Aldrin	-30	0.986	74	71	90	18	9	6	10	18	12	7	11	0.03	0.08	31	13
	Dieldrin	12	0.988	91	98	95	17	8	10	11	17	9	13	11	0.01	0.03	36	16
	Endrin	2	0.988	93	87	91	12	5	6	5	15	6	8	12	0.009	0.03	39	11
	α-endosulfan	-30	0.987	70	77	97	7	16	7	11	10	17	10	11	0.03	0.08	37	24
	β-endosulfan	-28	0.986	81	83	84	8	12	12	16	9	17	13	16	0.03	0.08	33	17
	DDE	-19	0.987	69	76	88	9	13	8	2	11	13	11	4	0.001	0.003	30	24
	DDT	-17	0.986	68	77	94	19	8	14	3	20	11	17	10	0.03	0.08	30	23
	DDD	-16	0.985	65	74	97	17	9	7	5	19	11	11	11	0.006	0.02	25	18
	Methoxychlor	-4	0.987	70	72	90	13	10	4	8	15	11	14	9	0.01	0.03	22	20

DDD - dichlorodiphenyldichloroethane; DDE - 2,2-bis(p-chlorophenyl)-1,1-dichloroethene; DDT - dichlorodiphenyltrichloroethane; HCB - hexachlorobenzene; HCH - hexachlorocyclohexane; MDL – method detection limit; MQL – method quantification limit; OCP – organochlorine pesticide; *Ur,tot* - expanded combined uncertainty.

Table S3. Method validation parameters for SM in breast adipose tissue spiked at different levels.

	Analyte	Ion enhancement/ suppression (%)	Coefficient of determination	MDL (μg/g)	MQL (μg/g)
SM	ABDI	20	0.988	0.01	0.04
	MA	11	0.990	0.03	0.08
	HHCB	-4	0.987	0.009	0.03
	AHTN	-8	0.988	0.005	0.02
	MK	23	0.999	0.01	0.04
	MX	-26	0.987	0.01	0.03

ABDI – celestolide; AHTN – tonalide; HHCB – galaxolide; MA – musk ambrette; MDL – method detection limit; MK – musk ketone; MQL – method quantification limit; MX – musk xylene; SM – synthetic musks.

Table S4. Method validation parameters for PCB, BFR, OPP, OPE, and PAH in breast adipose tissue spiked at different levels.

	Analyte	Coefficient of determination	MDL (μg/g)	MQL (μg/g)
PCB	PCB 28	0.993	0.01	0.04
	PCB 52	0.992	0.02	0.05
	PCB 101	0.991	0.02	0.06
	PCB 77	0.994	0.01	0.03
	PCB 118	0.994	0.009	0.03
	PCB 114	0.989	0.008	0.02
	PCB 153	0.989	0.01	0.04
	PCB 138	0.987	0.01	0.03
	PCB 126	0.993	0.004	0.01
	PCB 156	0.991	0.007	0.02
	PCB 157	0.993	0.01	0.04
	PCB 180	0.991	0.002	0.005
	PCB 169	0.992	0.01	0.04
BFR	BDE 28	0.996	0.002	0.005
	PBT	0.992	0.04	0.1
	PBEB	0.996	0.04	0.1
	BDE 47	0.993	0.01	0.04
	BDE 100	0.997	0.01	0.03
	BDE 99	0.994	0.02	0.04
	TBB	0.995	0.01	0.03
	BDE 154	0.990	0.02	0.07
	BDE 153	0.993	0.009	0.03
	BDE 183	0.992	0.02	0.07
OPP	Dimethoate	0.987	0.009	0.03
	Chlorpyrifos-methyl	0.986	0.002	0.007
	Parathion-methyl	0.986	0.002	0.007
	Malathion	0.987	0.006	0.02
	Chlorpyrifos	0.988	0.006	0.02
	Chlorfenvinphos	0.988	0.005	0.01
OPE	TPrP	0.992	0.001	0.004
	TiBP	0.988	0.005	0.02
	TnBP	0.988	0.006	0.02
	TCEP	0.986	0.002	0.005
	TBEP	0.987	0.005	0.02
	TPhP	0.966	0.006	0.02
	TEHP	0.988	0.003	0.01
	TCP	0.985	0.001	0.003

BDE – bromodiphenyl ether; BFR – brominated flame retardant; MDL – method detection limit; MQL – method quantification limit; OPE - organophosphorus ester; OPP - organophosphorus pesticide; PBEB – pentabromoethylbenzene; PBT – pentabromotoluene; PCB - polychlorinated biphenyl; TBB - 2-ethylhexyl 2,3,4,5-tetrabromobenzoate; TBEP - tris(2-butoxyethyl) phosphate; TCEP - tris(2-chloroethyl) phosphate; TCP - tri-o-tolyl phosphate or tri-o-cresyl phosphate; TEHP - tris(2-ethylhexyl) phosphate; TiBP - tri-iso-butyl phosphate; TnBP - tri-n-butyl phosphate; TPrP - tripropyl phosphate

Table S4. Method validation parameters for PCB, BFR, OPP, OPE and PAH in breast adipose tissue spiked at different levels (continuation).

	Analyte	Coefficient of determination	MDL (µg/g)	MQL (µg/g)
PAH	Naph	0.992	0.01	0.03
	Acy	0.993	0.02	0.07
	Ace	0.993	0.01	0.03
	Flu	0.992	0.002	0.007
	Phe	0.991	0.001	0.004
	Ant	0.991	0.001	0.004
	Fln	0.991	0.002	0.007
	Pyr	0.994	0.0009	0.003
	B(a)A	0.991	0.001	0.004
	Chry	0.993	0.001	0.003
	B(b)Ft+ B(j)Ft	0.991	0.005	0.02
	B(k)Ft	0.991	0.001	0.004
	B(a)P	0.991	0.001	0.004
	DB(a,L)P	0.992	0.002	0.007
	DB(a,h)A	0.993	0.002	0.007
	B(g,h,i)P	0.990	0.002	0.008
	InP	0.991	0.001	0.004

Ace – acenaphthene; Acy – acenaphthylene; Ant – anthracene; B[a]A - benz[a]anthracene; B[a]P - benzo[a]pyrene; B[b]Ft - benzo[b]fluoranthene; B[g,h,i]P - benzo[g,h,i]perylene; B[j]Ft - benzo[j]fluoranthene; B[k]Ft - benzo[k]fluoranthene; Chry – chrysene; DB[a,h]A - dibenz[a,h]anthracene; DB[a,l]P - dibenzo[a,l]pyrene; Fln – fluoranthene; Flu – fluorene; InP - indeno[1,2,3-cd]pyrene; MDL – method detection limit; MQL – method quantification limit; Naph – naphthalene; PAH - polycyclic aromatic hydrocarbon; Phe – phenanthrene; Pyr – pyrene.

Table S5. SM, PCB, BFR, OCP, OPP, OPE, and PAH concentrations in cases and controls (μg/g breast adipose tissue).

Analyte	Case				Control				Case vs Control
	n	Frequency	Median (μg/g AT)	IQR	n	Frequency	Median (μg/g AT)	IQR	*p*
ABDI	0		nd		0		nd		
MA	2	4.8%	0.4	0.7	1	16.7%	0.1	na	1
HHCB	42	100%	0.2	0.3	6	100%	0.2	0.1	0.217
AHTN	39	92.9%	0.05	0.08	6	100%	0.05	0.03	0.503
MX	0		nd		0		nd		
MK	0		nd		0		nd		
ΣSM	42	100%	0.2	0.3	6	100%	0.3	0.2	0.170
PCB 28	0		nd		0		nd		
PCB 52	0		nd		0		nd		
PCB 101	0		nd		0		nd		
PCB 77	0		nd		0		nd		
PCB 118	0		nd		0		nd		
PCB 114	0		nd		0		nd		
PCB 153	31	73.8%	<MDL	na	6	100%	<MDL	na	na
PCB 138	14	33.3%	<MDL	na	4	66.7%	<MDL	na	na
PCB 126	0		nd		0		nd		
PCB 156	1	2.4%	<MDL	na	0		nd		na
PCB 157	0		nd		0		nd		
PCB 180	7	16.7%	0.05	0.02	2	33.3%	0.05	0.01	0.442
PCB 169	0		nd		0		nd		
ΣPCB	31	73.8%	0.02	0.01	6	100%	0.02	0.05	0.296
BDE 28	0		nd		0		nd		
PBT	0		nd		0		nd		
PBEB	0		nd		0		nd		
BDE 47	0		nd		0		nd		
BDE 100	0		nd		0		nd		
BDE 99	0		nd		0		nd		
TBB	0		nd		0		nd		
BDE 154	0		nd		0		nd		
BDE 153	0		nd		0		nd		
BDE 183	0		nd		0		nd		
ΣBFR	0		nd		0		nd		
α-HCH	0		nd		0		nd		
β-HCH	0		nd		0		nd		
γ-HCH	1	2.4%	<MDL	na	0		nd		na
δ-HCH	0		nd		0		nd		
HCB	3	7.1%	0.05	0.11	1	16.7%	0.2	na	0.143
Aldrin	0		nd		0		nd		
Dieldrin	0		nd		0		nd		
Endrin	0		nd		0		nd		
α-endosulfan	0		nd		0		nd		
β-endosulfan	0		nd		0		nd		
DDE	25	59.5%	0.08	0.22	6	100%	<MDL	na	0.051
DDD	0		nd		0		nd		
DDT	0		nd		0		nd		
Methoxychlor	0		nd		0		nd		
ΣOCP	27	64.3%	0.08	0.19	6	100%	0.0006	0.21	0.306
Dimethoate	0		nd		0		nd		
Chlorpyrifos-methyl	0		nd		0		nd		
Parathion-methyl	0		nd		0		nd		
Malathion	0		nd		0		nd		
Chlorpyrifos	0		nd		0		nd		
Chlorfenvinphos	0		nd		0		nd		
ΣOPP	0		nd		0		nd		

ABDI – celestolide; AHTN – tonalide; BDE – bromodiphenyl ether; BFR – brominated flame retardant; DDD - dichlorodiphenyldichloroethane; DDE - 2,2-bis(p-chlorophenyl)-1,1-dichloroethene; DDT -

dichlorodiphenyltrichloroethane; ED – endocrine disruptor; HCB - hexachlorobenzene; HCH - hexachlorocyclohexane; HHCB – galaxolide; IQR – interquartile range; MA – musk ambrette; MDL – method detection limit; MK – musk ketone; MQL – method quantification limit; MX – musk xylene; na – not applicable; nd – not detected; OPP - organophosphorus pesticide; PBEB – pentabromoethylbenzene; PBT – pentabromotoluene; PCB - polychlorinated biphenyl; TBB - 2-ethylhexyl 2,3,4,5-tetrabromobenzoate. Statistical analysis performed with Mann-Whitney and Kruskal Wallis tests, $p < 0.05$.

Table S5. SM, PCB, BFR, OCP, OPP, OPE, and PAH concentrations in cases and controls (μg/g breast adipose tissue) (continuation).

Analyte	Case				Control				Case vs Control
	**n**	**Frequency**	**Median (μg/g AT)**	**IQR**	**n**	**Frequency**	**Median (μg/g AT)**	**IQR**	***p***
TPrP	0		nd		0		nd		
TiBP	39	92.9%	0.046	0.003	6	100%	0.048	0.001	**0.005**
TnBP	6	14.3%	0.042	0.002	1	16.7%	0.05	na	0.088
TCEP	0		nd		0		nd		
TBEP	8	19.0%	0.04	0.02	0		nd		na
TPhP	13	31.0%	0.057	0.003	0		nd		na
TEHP	0		nd		0		nd		
TCP	42	100%	<MDL	na	6	100%	<MDL	na	0.275
ΣOPE	42	100%	0.08	0.06	6	100%	0.05	0.02	0.308
Naph	36	85.7%	<MDL	na	4	66.7%	<MQL	0.05	**0.01**
Acy	12	28.6%	<MDL	na	0		nd		na
Ace	34	81.0%	<MDL	na	0		nd		na
Flu	42	100%	<MDL	na	6	100%	<MDL	na	1
Phe	42	100%	<MDL	na	6	100%	<MDL	na	1
Ant	40	95.2%	<MDL	na	6	100%	<MDL	na	1
Fln	3	7.1%	<MDL	na	0		nd		na
Pyr	12	28.6%	<MDL	na	1	16.7%	<MDL	na	1
B(a)A	0		nd		0		nd		
Chry	0		nd		0		nd		
B(b)Ft+ B(j)Ft	0		nd		0		nd		
B(k)Ft	0		nd		0		nd		
B(a)P	0		nd		0		nd		
DB(a,L)P	0		nd		0		nd		
DB(a,h)A	0		nd		0		nd		
B(g,h,i)P	0		nd		0		nd		
InP	0		nd		0		nd		
ΣPAH	42	100%	0.02	0.01	6	100%	0.02	0.02	0.635
ΣED	42	100%	0.4	0.5	6	100%	0.5	0.3	0.184

Ace – acenaphthene; Acy – acenaphthylene; Ant – anthracene; B[a]A - benz[a]anthracene; B[a]P - benzo[a]pyrene; B[b]Ft - benzo[b]fluoranthene; B[g,h,i]P - benzo[g,h,i]perylene; B[j]Ft - benzo[j]fluoranthene; B[k]Ft - benzo[k]fluoranthene; Chry – chrysene; DB[a,h]A - dibenz[a,h]anthracene; DB[a,l]P - dibenzo[a,l]pyrene; ED – endocrine disruptor; Fln – fluoranthene; Flu – fluorene; InP - indeno[1,2,3-cd]pyrene; IQR – interquartile range; MDL – method detection limit; MQL – method quantification limit; na – not applicable; Naph – naphthalene; nd – not detected; OPE - organophosphorus ester; PAH - polycyclic aromatic hydrocarbon; Phe – phenanthrene; Pyr – pyrene; TBEP - tris(2-butoxyethyl) phosphate; TCEP - tris(2-chloroethyl) phosphate; TCP - tri-o-tolyl phosphate or tri-o-cresyl phosphate; TEHP - tris(2-ethylhexyl) phosphate; TiBP - tri-iso-butyl phosphate; TnBP - tri-n-butyl phosphate; TPrP - tripropyl phosphate. Statistical analysis performed with Mann-Whitney and Kruskal Wallis tests, $p < 0.05$.

Table S6. SM, PCB, BFR, OCP, OPP, OPE, and PAH concentrations in hormonal and non-hormonal breast cancer (μg/g breast adipose tissue).

Analyte	Hormonal Breast Cancer				Non-hormonal Breast Cancer				Hormonal vs Non-hormonal Breast Cancer
	n	Frequency	Median (μg/g AT)	IQR	n	Frequency	Median (μg/g AT)	IQR	*p*
ABDI	0		nd		0		nd		
MA	1	5.0%	0.7	na	1	16.7%	<MQL	na	0.333
HHCB	20	100%	0.1	0.2	6	100%	0.2	0.3	0.213
AHTN	20	100%	0.05	0.08	5	83.3%	0.06	0.09	0.676
MX	0		nd		0		nd		
MK	0		nd		0		nd		
ΣSM	20	100%	0.1	0.2	6	100%	0.3	0.4	0.232
PCB 28	0		nd		0		nd		
PCB 52	0		nd		0		nd		
PCB 101	0		nd		0		nd		
PCB 77	0		nd		0		nd		
PCB 118	0		nd		0		nd		
PCB 114	0		nd		0		nd		
PCB 153	16	77.5%	<MDL	na	5	83.3%	<MDL	na	1
PCB 138	7	35.0%	<MDL	na	4	66.7%	<MDL	na	1
PCB 126	0		nd		0		nd		
PCB 156	1	5.0%	<MDL	na	0		nd		na
PCB 157	0		nd		0		nd		
PCB 180	5	25.0%	0.05	0.02	1	16.7%	0.04	na	0.273
PCB 169	0		nd		0		nd		
ΣPCB	16	80.0%	0.02	0.04	5	83.3%	0.02	0.01	0.390
BDE 28	0		nd		0		nd		
PBT	0		nd		0		nd		
PBEB	0		nd		0		nd		
BDE 47	0		nd		0		nd		
BDE 100	0		nd		0		nd		
BDE 99	0		nd		0		nd		
TBB	0		nd		0		nd		
BDE 154	0		nd		0		nd		
BDE 153	0		nd		0		nd		
BDE 183	0		nd		0		nd		
ΣPCB	0		nd		0		nd		
α-HCH	0		nd		0		nd		
β-HCH	0		nd		0		nd		
γ-HCH	1	5.0%	<MDL	na	0		nd		
δ-HCH	0		nd		0		nd		
HCB	2	10.0%	0.05	0.01	0		nd		
Aldrin	0		nd		0		nd		
Dieldrin	0		nd		0		nd		
Endrin	0		nd		0		nd		
α-endosulfan	0		nd		0		nd		
β-endosulfan	0		nd		0		nd		
DDE	12	60.0%	0.1	0.2	5	83.3%	<MDL	0.12	0.381
DDD	0		nd		0		nd		
DDT	0		nd		0		nd		
Methoxychlor	0		nd		0		nd		
ΣOCP	13	65.0%	0.1	0.2	5	83.3%	0.0006	0.1	0.355
Dimethoate	0		nd		0		nd		
Chlorpyrifos-methyl	0		nd		0		nd		
Parathion-methyl	0		nd		0		nd		
Malathion	0		nd		0		nd		
Chlorpyrifos	0		nd		0		nd		
Chlorfenvinphos	0		nd		0		nd		
ΣOPP	0		nd		0		nd		

ABDI – celestolide; AHTN – tonalide; BDE – bromodiphenyl ether; BFR – brominated flame retardant; DDD - dichlorodiphenyldichloroethane; DDE - 2,2-bis(p-chlorophenyl)-1,1-dichloroethene; DDT - dichlorodiphenyltrichloroethane; ED – endocrine disruptor; HCB - hexachlorobenzene; HCH - hexachlorocyclohexane; HHCB – galaxolide; IQR – interquartile range; MA – musk ambrette; MDL – method detection limit; MK – musk ketone; MQL – method quantification limit; MX – musk xylene; na – not applicable; nd – not detected; OPP - organophosphorus pesticide; PBEB – pentabromoethylbenzene; PBT – pentabromotoluene; PCB - polychlorinated biphenyl; TBB - 2-ethylhexyl 2,3,4,5-tetrabromobenzoate. Statistical analysis performed with Mann-Whitney and Kruskal Wallis tests, $p < 0.05$.

Table S6. SM, PCB, BFR, OCP, OPP, OPE, and PAH concentrations in hormonal and non-hormonal breast cancer (μg/g breast adipose tissue) (continuation).

Analyte	Hormonal Breast Cancer				Non-hormonal Breast Cancer				Hormonal vs Non-hormonal Breast Cancer
	n	Frequency	Median (μg/g AT)	IQR	n	Frequency	Median (μg/g AT)	IQR	*p*
TPrP	0		nd		0		nd		
TiBP	20	100%	0.046	0.004	4	66.7%	0.045	0.002	0.174
TnBP	4	20.0%	0.04	0.02	0		nd		na
TCEP	0		nd		0		nd		
TBEP	5	25.0%	0.05	0.03	1	16.7%	0.06	na	0.485
TPhP	6	30.0%	0.057	0.005	2	33.3%	0.057	0.005	0.521
TEHP	0		nd		0		nd		
TCP	20	100%	0.0007	na	6	100%	0.0007	na	0.729
ΣOPE	20	100%	0.09	0.06	6	100%	0.05	0.06	0.128
Naph	18	90.0%	0.007	0.020	6	100%	<MDL	na	**0.044**
Acy	7	35.0%	0.01	na	1	16.7%	<MDL	na	0.817
Ace	14	70.0%	<MDL	na	5	83.3%	<MDL	na	1
Flu	20	100%	<MDL	na	6	100%	<MDL	na	1
Phe	20	100%	<MDL	na	6	100%	<MDL	na	1
Ant	19	95.0%	<MDL	na	6	100%	<MDL	na	1
Fln	1	5.0%	<MDL	na	2	33.3%	<MDL	na	1
Pyr	8	40.0%	<MDL	na	3	50.0%	<MDL	na	1
B(a)A	0		nd		0		nd		
Chry	0		nd		0		nd		
B(b)Ft+ B(j)Ft	0		nd		0		nd		
B(k)Ft	0		nd		0		nd		
B(a)P	0		nd		0		nd		
DB(a,L)P	0		nd		0		nd		
DB(a,h)A	0		nd		0		nd		
B(g,h,i)P	0		nd		0		nd		
InP	0		nd		0		nd		
ΣPAH	20	100%	0.02	0.02	6	100%	0.018	0.002	0.295
ΣED	20	100%	0.3	0.4	6	100%	0.5	0.3	0.447

Ace – acenaphthene; Acy – acenaphthylene; Ant – anthracene; B[a]A - benz[a]anthracene; B[a]P - benzo[a]pyrene; B[b]Ft - benzo[b]fluoranthene; B[g,h,i]P - benzo[g,h,i]perylene; B[j]Ft - benzo[j]fluoranthene; B[k]Ft - benzo[k]fluoranthene; Chry – chrysene; DB[a,h]A - dibenz[a,h]anthracene; DB[a,l]P - dibenzo[a,l]pyrene; ED – endocrine disruptor; Fln – fluoranthene; Flu – fluorene; InP - indeno[1,2,3-cd]pyrene; IQR – interquartile range; MDL – method detection limit; MQL – method quantification limit; na – not applicable; Naph – naphthalene; nd – not detected; OPE - organophosphorus ester; PAH - polycyclic aromatic hydrocarbon; Phe – phenanthrene; Pyr – pyrene; TBB - 2-ethylhexyl 2,3,4,5-tetrabromobenzoate; TBEP - tris(2-butoxyethyl) phosphate; TCEP - tris(2-chloroethyl) phosphate; TCP - tri-o-tolyl phosphate or tri-o-cresyl phosphate; TEHP - tris(2-ethylhexyl) phosphate; TiBP - tri-iso-butyl phosphate; TnBP - tri-n-butyl phosphate; TPrP - tripropyl phosphate. Statistical analysis performed with Mann-Whitney and Kruskal Wallis tests, $p < 0.05$.

Table S7. Total lipids concentration (g/g breast adipose tissue) and fatty acids profile in hormonal and non-hormonal breast cancer (%).

	Hormonal Breast Cancer				Non- hormonal Breast Cancer				Hormonal vs Non-hormonal breast cancer
	**n**	**Frequency**	**Median**	**IQR**	**n**	**Frequency**	**Median**	**IQR**	***p***
Total Lipids	20	100%	1.0	0.1	6	100%	0.9	0.2	0.693
**Fatty acids**									
C4:0	0		nd		0		nd		
C6:0	0		nd		0		nd		
C8:0	0		nd		0		nd		
C10:0	20	100%	0.02	0.02	6	100%	0.01	0.02	0.587
C11:0	0		nd		0		nd		
C12:0	20	100%	0.3	0.1	6	100%	0.3	0.2	0.914
C14:0	20	100%	2.2	0.8	6	100%	2.1	0.5	0.828
C15:0	20	100%	0.20	0.06	6	100%	0.17	0.02	0.123
C16:0	20	100%	23	3	6	100%	23	3	0.434
C17:0	20	100%	0.22	0.04	6	100%	0.21	0.04	0.278
C18:0	20	100%	4	2	6	100%	4.0	0.5	0.056
C20:0	20	100%	0.13	0.06	6	100%	0.10	0.03	0.297
C21:0	0		nd		0		nd		
C22:0	20	100%	0.03	0.02	6	100%	0.018	0.006	0.165
C23:0	0		nd		0		nd		
C24:0	0		nd		0		nd		
ΣSFA	20	100%	31	5	6	100%	31	3	0.373
C14:1 ω5	20	100%	0.11	0.07	6	100%	0.14	0.08	0.09
C15:1 ω5 *cis*	0		nd		0		nd		
C16:1 ω7	20	100%	3	1	6	100%	3	1	0.664
C17:1 ω7 *cis*	0		nd		0		nd		
C18:1 ω9 *trans*	0		nd		0		nd		
C18:1 ω9 *cis*	20	100%	46	7	6	100%	47	8	0.543
C20:1 ω9 *cis*	20	100%	0.6	0.1	6	100%	0.7	0.4	0.745
C22:1 ω9	20	100%	0.023	0.008	6	100%	0.024	0.008	**0.04**
C24:1 ω9	8	40%	0.013	0.005	3	50%	0.009	0.005	**0.04**
ΣMUFA	20	100%	50	5	6	100%	52	6	0.474
C18:2 ω6 *trans*	0		nd		0		nd		
C18:2 ω6 *cis*	20	100%	17	4	6	100%	16	3	0.587
C18:3 ω3	20	100%	0.5	0.2	6	100%	0.5	0.6	0.948
C18:3 ω6	20	100%	0.04	0.02	6	100%	0.04	0.03	0.086
C20:2 ω6 *cis*	20	100%	0.3	0.1	6	100%	0.27	0.08	0.287
C20:3 ω3 *cis*	20	100%	0.017	0.006	6	100%	0.019	0.008	0.307
C20:3 ω6 *cis*	20	100%	0.3	0.2	6	100%	0.4	0.2	0.259
C20:4 ω6	20	100%	0.5	0.2	6	100%	0.6	0.4	0.103
C20:5 ω3 *cis*	20	100%	0.12	0.07	6	100%	0.13	0.07	0.914
C22:2 ω6 *cis*	0		nd		0		nd		
C22:6 ω3 *cis*	20	100%	0.4	0.2	6	100%	0.6	0.4	0.373
ΣPUFA	20	100%	19	4	6	100%	19	3	**0.03**
Σω3	20	100%	1.1	0.3	6	100%	1.3	0.3	**0.02**
Σω6	20	100%	18	4	6	100%	17	3	**0.03**

IQR – interquartile range; MUFA – monounsaturated fatty acids; PUFA – polyunsaturated fatty acids; SFA – saturated fatty acids; na – not applicable; nd – not detected; ω – omega; ω3– omega 3 fatty acids; ω6 – omega 6 fatty acids; Statistical analysis performed with Mann-Whitney and Kruskal Wallis tests, $p < 0.05$; Significant *p* values are shown in bold.

Table S8. Spearman's correlation coefficient (rS) and *p* values between endocrine disruptors and the fatty acid profile in breast adipose tissue from breast cancer patients. The values in bold indicate significant associations (*p*<0.05).

		ΣSM		ΣPCB		ΣOCP		ΣOPE		ΣPAH		ΣED	
		rs	*p*	rs	*p*	rs	*p*	rs	*p*	rs	*p*	rs	*p*
							All cases						
	Total lipids	-0.11	0.332	-0.07	0.572	-0.06	0.686	**0.28**	**0.011**	**-0.30**	**0.005**	-0.10	0.374
SFA	C10:0	-0.02	0.877	0.21	0.116	0.12	0.373	-0.12	0.284	**0.30**	**0.007**	0.03	0.777
	C12:0	0.07	0.541	**0.36**	**0.004**	0.23	0.096	-0.08	0.446	0.13	0.231	0.13	0.238
	C14:0	-0.21	0.051	0.03	0.821	-0.14	0.318	-0.07	0.537	0.14	0.213	-0.20	0.064
	C15:0	**-0.23**	**0.034**	-0.09	0.494	**-0.37**	**0.006**	-0.21	0.052	0.20	0.076	**-0.30**	**0.005**
	C16:0	-0.13	0.229	0.12	0.354	**-0.29**	**0.035**	-0.05	0.680	-0.10	0.383	-0.21	0.059
	C17:0	-0.07	0.534	**0.27**	**0.032**	-0.26	0.054	-0.14	0.199	**0.41**	**<0.0001**	-0.10	0.349
	C18:0	-0.06	0.573	**0.26**	**0.042**	0.09	0.509	0.11	0.331	0.15	0.174	-0.04	0.755
	C20:0	-0.07	0.527	0.20	0.119	0.22	0.106	0.19	0.076	0.01	0.931	0.00	1.000
	C22:0	0.00	0.985	0.09	0.468	0.15	0.269	0.16	0.146	0.02	0.847	0.04	0.754
MUFA	C14:1ω5	0.09	0.408	0.06	0.662	-0.23	0.093	**-0.22**	**0.042**	0.05	0.635	0.06	0.613
	C16:1ω7	0.17	0.125	0.02	0.871	-0.26	0.062	-0.18	0.101	-0.01	0.961	0.14	0.212
	C18:1ω9 *cis*	0.03	0.804	-0.08	0.547	0.17	0.212	0.13	0.259	-0.08	0.470	0.08	0.468
	C20:1ω9	-0.10	0.344	**-0.33**	**0.008**	-0.01	0.932	0.02	0.878	0.09	0.422	-0.13	0.246
	C22:1ω9	-0.05	0.655	**0.26**	**0.038**	0.24	0.078	-0.05	0.662	**0.31**	**0.005**	0.07	0.527
	C24:1ω9	0.08	0.647	0.09	0.675	0.29	0.175	0.12	0.487	**0.37**	**0.020**	0.24	0.143
PUFA	C18:2ω6 *cis*	0.03	0.771	-0.04	0.739	0.07	0.611	0.04	0.701	-0.01	0.907	-0.01	0.948
	C18:3ω3	0.12	0.273	0.12	0.375	0.03	0.829	-0.10	0.346	**0.33**	**0.002**	0.16	0.139
	C18:3ω6	0.18	0.107	0.20	0.115	0.20	0.141	**-0.23**	**0.034**	0.04	0.737	0.20	0.075
	C20:2ω6	-0.13	0.255	-0.09	0.467	-0.08	0.582	-0.17	0.128	0.09	0.401	-0.17	0.127
	C20:3ω3	-0.09	0.425	0.24	0.063	-0.19	0.163	-0.15	0.163	**0.24**	**0.031**	-0.08	0.445
	C20:3ω6	0.05	0.631	-0.14	0.286	-0.12	0.398	-0.20	0.072	-0.10	0.345	-0.05	0.660
	C20:4ω6	0.11	0.340	-0.04	0.739	0.02	0.881	**-0.27**	**0.013**	0.00	0.998	0..27	0.806
	C20:5ω3	0.16	0.137	0.24	0.056	0.01	0.926	-0.07	0.505	**0.23**	**0.033**	**0.22**	**0.046**
	C22:6ω3	-0.01	0.955	-0.09	0.485	0.02	0.866	0.05	0.659	0.14	0.194	0.04	0.718
	ΣSFA	-0.15	0.179	0.21	0.098	-0.23	0.088	0.00	0.981	0.00	0.968	-0.20	0.065
	ΣMUFA	0.07	0.529	-0.07	0.590	0.07	0.604	0.09	0.396	-0.08	0.496	0.14	0.195
	ΣPUFA	0.01	0.906	-0.06	0.660	0.08	0.579	0.02	0.873	0.03	0.762	-0.02	0.848
	Σω3	0.05	0.675	-0.04	0.744	0.00	0.983	-0.05	0.673	0.21	0.057	0.10	0.374
	Σω6	0.03	0.803	-0.08	0.522	0.05	0.720	0.00	0.983	-0.01	0.943	-0.03	0.798

MUFA – monounsaturated fatty acids; PUFA – polyunsaturated fatty acids; rs - Spearman's correlation coefficient; SFA – saturated fatty acids; ω – omega; ω3– omega 3 fatty acids; ω6 – omega 6 fatty acids. Significant p values are shown in bold, $p < 0.05$.

Table S9. Spearman's correlation coefficient (rS) and *p* values between endocrine disruptors and the fatty acid profile in breast adipose tissue from controls. The values in bold indicate significant associations (*p*<0.05).

		ΣSM		ΣPCB		ΣOCP		ΣOPE		ΣPAH		ΣED	
		rs	*p*	rs	*p*	rs	*p*	rs	*p*	rs	*p*	rs	*p*
							**Control**						
	Total lipids	-0.45	0.142	-0.27	0.402	-0.02	0.960	0.06	0.843	-0.01	0.977	-0.42	0.172
SFA	C10:0	-0.14	0.665	**-0.75**	**0.005**	**-0.80**	**0.002**	0.04	0.897	-0.14	0.661	**-0.69**	**0.014**
	C12:0	-0.12	0.713	**-0.78**	**0.003**	**-0.82**	**0.001**	0.01	0.966	-0.12	0.711	**-0.67**	**0.017**
	C14:0	-0.15	0.633	-0.52	0.082	**-0.78**	**0.003**	0.25	0.443	-0.03	0.929	**-0.58**	**0.048**
	C15:0	0.24	0.457	0.03	0.929	-0.13	0.680	**0.77**	**0.003**	-0.32	0.305	0.18	0.572
	C16:0	**-0.64**	**0.024**	**-0.73**	**0.008**	-0.45	0.143	0.11	0.729	0.05	0.866	**-0.84**	**0.001**
	C17:0	0.45	0.145	-0.23	0.475	-0.57	0.055	0.43	0.159	0.02	0.955	0.20	0.542
	C18:0	0.30	0.342	-0.44	0.158	**-0.80**	**0.002**	-0.52	0.080	-0.17	0.596	-0.44	0.152
	C20:0	0.36	0.245	-0.15	0.645	**-0.72**	**0.008**	-0.53	0.075	0.04	0.902	-0.21	0.513
	C22:0	**0.58**	**0.048**	-0.17	0.589	**-0.72**	**0.008**	-0.53	0.075	0.28	0.379	0.11	0.729
MUFA	C14:1ω5	**-0.80**	**0.002**	-0.06	0.858	0.37	0.242	**0.64**	**0.026**	0.08	0.796	-0.32	0.319
	C16:1ω7	**-0.64**	**0.026**	0.03	0.929	0.37	0.242	**0.71**	**0.010**	0.29	0.366	-0.04	0.914
	C18:1ω9 *cis*	-0.08	0.812	**0.78**	**0.003**	**0.72**	**0.008**	-0.03	0.931	-0.30	0.341	0.22	0.499
	C20:1ω9	0.11	0.729	**0.65**	**0.021**	**0.82**	**0.001**	-0.11	0.729	0.11	0.745	**0.63**	**0.028**
	C22:1ω9	-0.02	0.948	-0.18	0.581	-0.28	0.388	0.30	0.342	0.16	0.628	-0.06	0.846
	C24:1ω9	0.46	0.151	-0.20	0.551	-0.42	0.197	-0.52	0.102	-0.56	0.073	-0.21	0.537
PUFA	C18:2ω6 *cis*	0.55	0.063	-0.53	0.077	**-0.83**	**0.001**	-0.48	0.118	0.20	0.534	-0.02	0.948
	C18:3ω3	0.32	0.319	-0.44	0.158	-0.20	0.542	0.11	0.729	0.03	0.937	0.21	0.513
	C18:3ω6	0.35	0.265	**0.82**	**0.001**	0.21	0.508	0.55	0.063	-0.48	0.115	0.37	0.236
	C20:2ω6	**0.59**	**0.042**	0.14	0.669	-0.02	0.959	-0.27	0.391	**0.64**	**0.024**	**0.79**	**0.002**
	C20:3ω3	0.38	0.226	-0.03	0.920	0.19	0.551	0.00	1.000	-0.08	0.805	0.42	0.175
	C20:3ω6	0.52	0.080	0.49	0.103	0.27	0.403	0.50	0.095	-0.08	0.796	**0.76**	**0.005**
	C20:4ω6	-0.42	0.175	0.55	0.063	**0.80**	**0.002**	**0.61**	**0.036**	0.05	0.866	0.29	0.354
	C20:5ω3	-0.33	0.297	0.24	0.461	**0.74**	**0.006**	0.50	0.095	0.04	0.902	0.31	0.331
	C22:6ω3	0.47	0.124	0.50	0.098	0.28	0.373	0.55	0.063	-0.08	0.796	**0.73**	**0.007**
	ΣSFA	-0.43	0.167	**-0.84**	**0.001**	**-0.63**	**0.027**	0.01	0.983	-0.09	0.779	**-0.85**	**0.001**
	ΣMUFA	-0.52	0.080	**0.58**	**0.048**	**0.82**	**0.001**	0.32	0.319	-0.26	0.418	-0.02	0.948
	ΣPUFA	0.48	0.118	**-0.59**	**0.045**	**-0.74**	**0.006**	-0.37	0.236	0.39	0.212	0.08	0.795
	Σω3	0.41	0.183	-0.17	0.837	0.07	0.837	0.08	0.795	-0.14	0.661	0.37	0.236
	Σω6	0.53	0.075	**-0.59**	**0.045**	**-0.83**	**0.001**	-0.34	0.276	0.26	0.418	0.01	0.966

MUFA – monounsaturated fatty acids; PUFA – polyunsaturated fatty acids; rs - Spearman's correlation coefficient; SFA – saturated fatty acids; ω – omega; ω3– omega 3 fatty acids; ω6 – omega 6 fatty acids. Significant p values are shown in bold, *p* < 0.05.

Table S10. Spearman's correlation coefficient (rS) and *p* values between endocrine disruptors and the fatty acid profile in breast adipose tissue from hormonal breast cancer patients. The values in bold indicate significant associations (*p*<0.05).

		ΣSM		ΣPCB		ΣOCP		ΣOPE		ΣPAH		ΣED	
		rs	*p*	rs	*p*	rs	*p*	rs	*p*	rs	*p*	rs	*p*
		**Hormonal breast cancer**											
	Total lipids	0.12	0.449	0.15	0.413	0.37	0.065	0.00	0.999	-0.14	0.379	0.14	0.393
SFA	C10:0	-0.16	0.336	0.08	0.664	**-0.41**	**0.035**	0.07	0.647	**0.38**	**0.017**	0.00	0.981
	C12:0	-0.13	0.429	**0.45**	**0.009**	-0.08	0.712	0.05	0.759	0.18	0.277	0.09	0.562
	C14:0	-0.20	0.207	0.02	0.920	**-0.50**	**0.009**	0.01	0.965	0.17	0.307	-0.12	0.479
	C15:0	-0.22	0.171	0.13	0.467	**-0.55**	**0.003**	-0.08	0.608	0.26	0.101	-0.14	0.387
	C16:0	-0.15	0.347	0.21	0.248	-0.34	0.090	0.00	0.989	-0.13	0.430	-0.12	0.447
	C17:0	-0.25	0.113	0.26	0.156	**-0.44**	**0.024**	0.06	0.692	**0.45**	**0.004**	-0.16	0.335
	C18:0	-0.27	0.089	0.11	0.537	0.13	0.523	**0.42**	**0.006**	-0.03	0.855	-0.10	0.547
	C20:0	-0.10	0.532	-0.04	0.815	0.34	0.087	**0.34**	**0.033**	-0.15	0.346	0.00	0.980
	C22:0	-0.09	0.582	-0.18	0.319	0.15	0.452	0.13	0.428	0.01	0.975	-0.11	0.515
MUFA	C14:1ω5	-0.03	0.850	0.06	0.739	**-0.51**	**0.007**	-0.16	0.337	0.30	0.059	0.03	0.844
	C16:1ω7	0.02	0.907	-0.05	0.806	**-0.48**	**0.014**	-0.16	0.309	0.29	0.066	0.02	0.881
	C18:1ω9 *cis*	0.05	0.747	-0.20	0.269	**0.46**	**0.017**	0.13	0.416	-0.17	0.307	0.11	0.501
	C20:1ω9	-0.22	0.182	**-0.54**	**0.001**	-0.08	0.699	0.04	0.807	-0.02	0.881	-0.29	0.072
	C22:1ω9	-0.27	0.097	0.05	0.787	0.18	0.388	-0.10	0.558	0.17	0.296	-0.07	0.686
	C24:1ω9	-0.15	0.587	0.01	0.981	0.35	0.293	0.12	0.660	-0.25	0.357	0.17	0.535
PUFA	C18:2ω6 *cis*	0.21	0.203	0.06	0.756	-0.14	0.491	-0.01	0.947	0.02	0.893	0.03	0.847
	C18:3ω3	-0.13	0.434	0.00	0.982	0.07	0.752	-0.08	0.638	**0.48**	**0.002**	-0.03	0.853
	C18:3ω6	**0.35**	**0.025**	**0.49**	**0.004**	0.19	0.355	-0.29	0.067	0.26	0.110	**0.35**	**0.027**
	C20:2ω6	-0.17	0.305	-0.13	0.482	-0.20	0.326	-0.10	0.547	-0.07	0.684	-0.19	0.252
	C20:3ω3	**-0.38**	**0.016**	0.31	0.087	0.09	0.672	-0.09	0.568	0.27	0.095	-0.12	0.472
	C20:3ω6	0.08	0.638	-0.03	0.880	-0.06	0.770	-0.24	0.134	-0.16	0.313	-0.03	0.850
	C20:4ω6	0.30	0.058	0.07	0.691	-0.05	0.803	-0.30	0.056	-0.05	0.771	0.17	0.298
	C20:5ω3	0.02	0.919	**0.44**	**0.013**	0.14	0.483	-0.04	0.823	0.28	0.075	0.19	0.252
	C22:6ω3	-0.01	0.975	0.09	0.610	0.08	0.689	0.29	0.067	0.01	0.937	0.15	0.343
	ΣSFA	-0.22	0.174	0.24	0.194	-0.37	0.062	0.14	0.381	-0.06	0.704	-0.13	0.433
	ΣMUFA	0.05	0.754	-0.22	0.222	0.22	0.287	0.02	0.918	0.04	0.789	0.10	0.528
	ΣPUFA	0.18	0.279	0.02	0.915	-0.20	0.319	-0.06	0.734	0.08	0.619	0.01	0.938
	Σω3	-0.16	0.330	0.04	0.837	0.10	0.641	0.03	0.852	**0.37**	**0.019**	0.03	0.857
	Σω6	0.21	0.187	-0.01	0.945	-0.26	0.198	-0.08	0.626	0.04	0.801	0.00	0.977

MUFA – monounsaturated fatty acids; PUFA – polyunsaturated fatty acids; rs - Spearman's correlation coefficient; SFA – saturated fatty acids; ω – omega; ω3– omega 3 fatty acids; ω6 – omega 6 fatty acids. Significant p values are shown in bold, $p < 0.05$.

Table S11. Spearman's correlation coefficient (rS) and *p* values between endocrine disruptors and the fatty acid profile in breast adipose tissue from non-hormonal breast cancer patients. The values in bold indicate significant associations (p<0.05).

		**ΣSM**		**ΣPCB**		**ΣOCP**		**ΣOPE**		**ΣPAH**		**ΣED**	
		rs	*p*	rs	*p*	rs	*p*	rs	*p*	rs	*p*	rs	*p*
		**Non-hormonal breast cancer**											
	Total lipids	-0.33	0.300	0.13	0.718	0.41	0.242	**0.75**	**0.005**	-0.23	0.471	-0.12	0.720
	C10:0	0.25	0.436	-0.34	0.340	0.31	0.377	-0.94	**<0.0001**	-0.09	0.790	0.08	0.812
	C12:0	0.57	0.053	-0.35	0.315	0.11	0.764	-0.94	**<0.0001**	-0.03	0.929	0.43	0.167
	C14:0	0.26	0.409	-0.35	0.315	0.20	0.570	-0.90	**<0.0001**	0.27	0.392	0.08	0.812
	C15:0	0.05	0.871	-0.62	0.054	-0.01	0.985	**-0.74**	**0.006**	0.04	0.912	-0.13	0.697
SFA	C16:0	0.22	0.491	0.34	0.340	0.17	0.637	-0.35	0.264	**0.80**	**0.002**	0.08	0.812
	C17:0	0.56	0.060	0.20	0.582	-0.56	0.092	**-0.68**	**0.016**	0.14	0.657	0.36	0.255
	C18:0	**0.77**	**0.003**	0.20	0.582	-0.16	0.665	**-0.84**	**0.0006**	-0.03	0.929	**0.63**	**0.028**
	C20:0	0.54	0.068	-0.05	0.887	-0.19	0.597	-0.49	0.103	-0.37	0.233	0.48	0.112
	C22:0	0.50	0.100	-0.21	0.565	-0.26	0.469	-0.50	0.097	-0.32	0.318	0.48	0.112
	C14:1ω5	**0.59**	**0.043**	0.35	0.315	-0.23	0.519	**-0.71**	**0.010**	**0.63**	**0.028**	0.42	0.175
	C16:1ω7	**0.75**	**0.005**	**0.68**	**0.029**	-0.23	0.519	**-0.59**	**0.043**	0.57	0.051	**0.60**	**0.039**
MUFA	C18:1ω9 *cis*	**-0.58**	**0.049**	-0.34	0.340	0.22	0.544	**0.70**	**0.011**	**-0.63**	**0.028**	-0.40	0.199
	C20:1ω9	-0.52	0.082	-0.34	0.340	**0.67**	**0.034**	0.54	0.068	0.11	0.723	-0.31	0.331
	C22:1ω9	-0.04	0.905	-0.22	0.532	0.46	0.176	-0.11	0.737	**0.70**	**0.011**	0.02	0.948
	C24:1ω9	0.58	0.228	0.00	1.000	-0.20	0.700	0.20	0.704	0.21	0.694	0.54	0.266
	C18:2ω6 *cis*	**0.63**	**0.028**	0.28	0.439	**-0.79**	**0.006**	-0.56	0.060	0.11	0.739	0.48	0.118
	C18:3ω3	0.21	0.505	-0.30	0.395	-0.60	0.066	-0.04	0.905	-0.37	0.233	0.25	0.430
	C18:3ω6	0.49	0.108	0.45	0.192	**-0.66**	**0.040**	-0.42	0.174	-0.16	0.624	0.32	0.308
	C20:2ω6	-0.06	0.854	-0.07	0.849	**0.68**	**0.030**	-0.07	0.837	**0.85**	**0.0005**	-0.01	0.983
PUFA	C20:3ω3	-0.42	0.174	-0.53	0.117	-0.51	0.136	0.09	0.787	-0.54	0.071	-0.48	0.118
	C20:3ω6	0.15	0.640	0.01	0.981	0.45	0.191	-0.02	0.957	**0.80**	**0.002**	0.25	0.430
	C20:4ω6	-0.21	0.519	0.45	0.192	**0.66**	**0.037**	0.22	0.491	0.29	0.366	-0.17	0.602
	C20:5ω3	-0.37	0.230	0.54	0.103	-0.10	0.778	**0.73**	**0.007**	0.16	0.624	-0.34	0.276
	C22:6ω3	**-0.81**	**0.001**	-0.35	0.315	0.22	0.544	**0.82**	**0.001**	-0.06	0.859	**-0.63**	**0.028**
	ΣSFA	0.50	0.097	-0.03	0.943	-0.14	0.693	**-0.84**	**0.0006**	0.44	0.148	0.30	0.342
	ΣMUFA	-0.54	0.073	-0.07	0.849	0.22	0.544	**0.83**	**0.0009**	-0.46	0.133	-0.34	0.286
	ΣPUFA	0.39	0.207	0.39	0.266	**-0.79**	**0.006**	0.02	0.948	0.19	0.547	0.37	0.236
	Σω3	-0.47	0.121	**-0.68**	**0.029**	-0.33	0.355	0.25	0.436	**-0.83**	**0.0008**	-0.41	0.191
	Σω6	**0.73**	**0.007**	0.62	0.054	**-0.77**	**0.010**	-0.42	0.178	0.36	0.252	**0.61**	**0.036**

MUFA – monounsaturated fatty acids; PUFA – polyunsaturated fatty acids; rs - Spearman's correlation coefficient; SFA – saturated fatty acids; ω – omega; ω3– omega 3 fatty acids; ω6 – omega 6 fatty acids. Significant p values are shown in bold, p < 0.05.

Table S12. Breast cancer patients' data and relation with the sum of SM, PCB, OCP, OPE, and PAH concentrations (µg/g breast adipose tissue).

			∑SM			∑PCB			∑OCP			∑OPE			∑PAH			∑ED		
**Characteristics**		**(%)**	**Median**	**IQR**	***p***	**Median**	**IQR**	***p***	**Median**	**IQR**	***p***	**Median**	**IQR**	***p***	**Median**	**IQR**	***p***	**Median**	**IQR**	***p***
**Area of residence**	Densely Populated	26	0.1	0.5	0.630	0.02	0.04	0.630	0.0006	0.09	**0.021**	0.06	0.06	0.617	0.02	0.02	0.970	0.3	0.3	0.739
	Moderately Populated	36	0.2	0.2		0.02	0.04		0.1	0.2		0.09	0.06		0.02	0.02		0.4	0.4	
**Surgeries (number)**	1	12	0.2	0.3	0.913	0.02	0.02	0.500	0.04	0.21	0.460	0.05	0.01	0.124	0.03	0.02	**0.004**	0.3	0.4	0.730
	2	10	0.2	0.3		0.01	0.06		0.001	0.140		0.10	0.08		0.03	0.01		0.4	0.3	
	3	2	0.2			0.1			nd			0.1			0.01			0.3		
	4	5	0.3	0.5		0.04	0.05		0.0006			0.08	0.15		0.012	0.002		0.5	0.3	
**Smoking**	Yes	7	0.1	0.2	0.400	0.06	0.06	0.106	0.05	0.09	0.407	0.10	0.10	0.676	0.02	0.05	0.689	0.3	0.1	0.705
	(Ex)smoker	7	0.3	1.0		0.01			0.0006			0.09	0.06		0.03	0.10		0.4	1.0	
	No	33	0.2	0.3		0.02	0.01		0.03	0.15		0.06	0.05		0.03	0.01		0.3	0.4	
**Overweight &Obese**	Yes	33	0.2	0.4	0.250	0.02	0.05	0.199	0.1	0.2	**0.006**	0.05	0.05	0.883	0.02	0.01	0.212	0.4	0.5	0.105
	No	14	0.1	0.2		0.02	0.01		0.0006	0.01		0.06	0.07		0.03	0.05		0.3	0.2	
**High blood pressure**	Yes	36	0.1	0.6	0.624	0.02	0.03	0.617	0.1	0.2	**0.002**	0.09	0.05	0.217	0.02	0.01	**0.018**	0.4	0.6	0.456
	No	21	0.2	0.2		0.02	0.04		0.0006	0.01		0.05	0.06		0.03	0.05		0.3	0.3	
**Hormone replacement therapy**	Yes	12	0.2	0.2	0.319	0.01	0.03	**0.040**	0.09	0.11	0.282	0.08	0.05	0.053	0.02	0.01	0.473	0.3	0.3	0.770
	No	17	0.1	0.2		0.02	0.05		0.04	0.09		0.10	0.06		0.02	0.03		0.3	0.2	
**Diabetes**	Yes	10	0.1	0.1	0.699	0.02	0.01	0.500	0.001	0.200	0.567	0.08	0.09	0.599	0.02	0.01	0.344	0.3	0.3	0.203
	No	48	0.2	0.5		0.02	0.04		0.06	0.12		0.07	0.05		0.02	0.02		0.4	0.4	
**Menopause**	Yes	33	0.1	0.2	0.892	0.02	0.01	1.000	0.08	0.11	0.828	0.07	0.06	0.206	0.02	0.02	0.855	0.3	0.4	0.892
	No	7	0.2	0.1		0.02	0.01		0.05			0.05	0.05		0.03	0.01		0.33	0.08	
**Prior chronic therapy**	Yes	45	0.2	0.5	**0.037**	0.02	0.03	0.116	0.04	0.15	0.775	0.07	0.05	0.699	0.02	0.01	0.171	0.4	0.3	0.102
	No	14	0.1	0.2		0.02	0.03		0.05	0.10		0.07	0.10		0.03	0.02		0.3	0.1	

ED – endocrine disruptor; IQR – interquartile range; OCP – organochlorine pesticide; OPE – organophosphate ester; PAH – polycyclic aromatic hydrocarbon; PCB - polychlorinated biphenyl; SM – synthetic musk; Statistical analysis performed with Mann-Whitney and Kruskal Wallis tests, $p < 0.05$; Significant p values are shown in bold.
